# Mother’s dietary quality during pregnancy and offspring’s dietary quality in adolescence: Follow-up from a national birth cohort study of 19,582 mother–offspring pairs

**DOI:** 10.1371/journal.pmed.1002911

**Published:** 2019-09-12

**Authors:** Anne Ahrendt Bjerregaard, Thorhallur Ingi Halldorsson, Inge Tetens, Sjurdur Frodi Olsen

**Affiliations:** 1 Center for Fetal Programming, Department of Epidemiology Research, Statens Serum Institut, Copenhagen, Denmark; 2 The Unit for Nutrition Research, Faculty of Food Science and Nutrition, School of Health Sciences, University of Iceland, Reykjavik, Iceland; 3 Vitality—Centre for Good Older Lives, Department of Nutrition, Exercise, and Sports, University of Copenhagen, Denmark; 4 Department of Nutrition, Harvard T. H. Chan School of Public Health, Boston, Massachusetts, United States of America; London School of Hygiene and Tropical Medicine, UNITED KINGDOM

## Abstract

**Background:**

The Developmental Origins of Health and Disease (DOHaD) hypothesis postulates that exposures during early life, such as maternal dietary intake during pregnancy, may have a lifelong impact on the individual’s susceptibility to diseases. The individual’s own lifestyle habits are obviously an additional factor, but we have only limited knowledge regarding how it may interact with prenatal exposures in determining later disease. To gain further insight into these potentially complex relationships, we examined the longitudinal association between maternal diet quality during pregnancy and diet quality in early adolescence in a contemporary cohort.

**Methods and findings:**

From 1996 to 2003, the Danish National Birth Cohort (DNBC) was established. Women from across the country were enrolled, and dietary intake in midpregnancy was assessed concurrently with a 360-item food frequency questionnaire (FFQ) (https://www.dnbc.dk/-/media/arkiv/projekt-sites/dnbc/kodeboeger/dnbc-food-frequency-questionnaire/dnbc-food-frequency-questionnaire-pdf.pdf?la=en). During 2013–2018, dietary intake was assessed at age 14 years with a 150-item FFQ (https://www.dnbc.dk/-/media/arkiv/projekt-sites/dnbc/kodeboeger/ffq-14/dnbc-ffq-14-english-translation.pdf?la=en) in the DNBC children. Among the 19,582 mother–offspring pairs included in the analyses, the mean age (±standard deviation [SD]) was 30.7 (±4.1) years and 14.0 (±0.0) years for mothers and offspring, respectively. The majority of both mothers (67%) and offspring (76%) were classified as normal weight. For both questionnaires, a Healthy Eating Index (HEI) was developed as an indicator for diet quality based on current Danish Food-Based Dietary Guidelines (FBDG) including eight components: fruits and vegetables, fish, dietary fibres, red meat, saturated fatty acids (SFAs), sodium, sugar-sweetened beverages (SSBs), and added sugar. The HEI score was divided into quartiles; individuals in the highest quartile represented those with the most optimal diet. The maternal HEI score was correlated positively with offspring HEI score (Pearson *r* = 0.22, *p* < 0.001). A log-linear binomial model was used to estimate the relative risk of the offspring being in the highest quartile of HEI at age 14 years if the mother was ranked in quartile 4 during pregnancy. Results showed that offspring born to mothers who were in the highest HEI quartile during pregnancy were more likely themselves to be located in the highest HEI quartile at age 14 years (risk ratio [RR]: 2.1, 95% confidence interval [CI]: 2.0, 2.3, *p* < 0.001). Adjusting for maternal prepregnancy body mass index (BMI), parity, education, alcohol intake, physical activity, smoking, and breastfeeding, as well as offspring total energy intake and sex, did not influence the effect estimates. The limitations of our study include that some attrition bias towards more healthy participants was observed when comparing participants with nonparticipants. Bias in the FFQ method may also have resulted in underrepresentation of adolescents with poorer diet quality.

**Conclusions:**

In this study using data from a large national birth cohort, we observed that maternal diet quality during pregnancy was associated with diet quality of the offspring at age 14 years. These findings indicate the importance of separating early dietary exposures from later dietary exposures when studying dietary aetiologies of diseases postulated to have developmental origins such as, for instance, obesity or asthma in observational settings.

## Abstract 2

Using a novel healthy eating index, Anne Ahrendt Bjerregaard and colleagues reveal a child has a healthier diet in adolescence if its mother did in pregnancy.

## Author summary

**Why was this study done?:**

- Maternal dietary intake during pregnancy may have a lifelong impact on the offspring’s susceptibility to diseases.
- There is limited knowledge regarding how the offspring’s own lifestyle habits may interact with prenatal exposures.

**What did the researchers do and find?:**

- The Danish National Birth Cohort was established to investigate exposures during pregnancy on mothers’ and offspring health.
- To our knowledge, it is the first large prospective cohort with extensive concurrently collected information on dietary habits from both pregnancy and adolescence.
- We developed a Healthy Eating Index (HEI) as an indicator, with higher scores indicating higher adherence to current evidence-based dietary guidelines.
- Analyses based on 19,582 mother–offspring pairs showed that a high-quality score in the mother strongly predicts a high-quality score in the offspring.

**What do these findings mean?:**

- The potential influence of maternal diet during pregnancy was observed at a time when offspring become increasingly independent and rely more on their own food preferences.
- Our results highlight the need for accounting for offspring’s own diet in studies aimed at examining maternal dietary habits in relation to later offspring health.
- Bias towards healthier participants should be taken into consideration, as well as the fact that the offspring completed the questionnaire themselves, which might have resulted in those with poorer diet quality being underrepresented in our study population.

## Introduction

Evidence from the field of Developmental Origins of Health and Disease (DOHaD) research has consolidated the hypothesis that certain dietary exposures during foetal life—i.e., certain components of the mother’s diet during pregnancy—can have a long-lasting impact on the child’s susceptibility to noncommunicable diseases [1–4]. Studying such relationships represents a significant challenge since they require many years of follow-up, leaving room for multiple factors to potentially contribute in a process that may eventually lead to disease. One such factor is the offspring’s own dietary habits [5, 6]. Prospective studies have, for example, found inverse and positive associations between intake of salt [7] during adolescence and blood pressure in adulthood. Other studies found an association between intake of sugar-sweetened beverages (SSBs) and obesity in children and adolescents, which is a strong risk factor for later development of type 2 diabetes [8]. Accordingly, a potential association between mother’s dietary habits during pregnancy and offspring’s dietary intake could exist, ultimately influencing later disease risk.

The use of dietary indices to evaluate whether overall dietary habits are ‘healthy’ has expanded as a results of inconclusive results when investigating individual foods or nutrients, which ignores the overall complexity of dietary intake [9–13]. Nevertheless, studies investigating a potential influence of prenatal dietary intake on offspring diet quality longitudinally using a diet quality index are limited. One study found a significant effect of maternal prenatal diet quality on 2- to 3-year–old offspring’s intake of fruits and vegetables, which, however seemed to be mediated by maternal postnatal diet [14]. Thus, it seems of interest to explore whether similar associations remain in later childhood, when the child becomes increasingly independent from their parents.

The Danish National Birth Cohort (DNBC) is, to our knowledge, one of the first large prospective studies to comprehensively assess dietary intake in pregnancy and again during the adolescent years. It thereby represents a unique research resource to disentangle the separate influences of prenatal versus later dietary exposures in relation to long-term health [15, 16]. Using dietary data assessed in midpregnancy among mothers and dietary data assessed among offspring at age 14 years did not require new ethical approval. The aim of the present study was to elucidate to what extent maternal diet quality during pregnancy is associated with diet quality in the offspring at age 14 years in the DNBC.

## Methods

### Study population

The DNBC was established between 1996 and 2003 and includes approximately 90,000 offspring from across the country. Women who could do interviews in Danish and completed a food frequency questionnaire (FFQ) (https://www.dnbc.dk/-/media/arkiv/projekt-sites/dnbc/kodeboeger/dnbc-food-frequency-questionnaire/dnbc-food-frequency-questionnaire-pdf.pdf?la=en) in midpregnancy (gestation week [GW] 25) were eligible for enrolment in the DNBC [15, 16]. Written informed consent was given by the mothers on behalf of themselves and their children. The DNBC was conducted according to the guidelines laid down in the Declaration of Helsinki, and all procedures involving human participants were approved by the National Committee on Health Research Ethics in Denmark (H-4-2011-045). A follow-up among children at age approximately 14 years (between 2012 and 2018, *n* = 36,064) focused on overall dietary intake using a comprehensive web-based FFQ (https://www.dnbc.dk/-/media/arkiv/projekt-sites/dnbc/kodeboeger/ffq-14/dnbc-ffq-14-english-translation.pdf?la=en).

**Fig 1:**
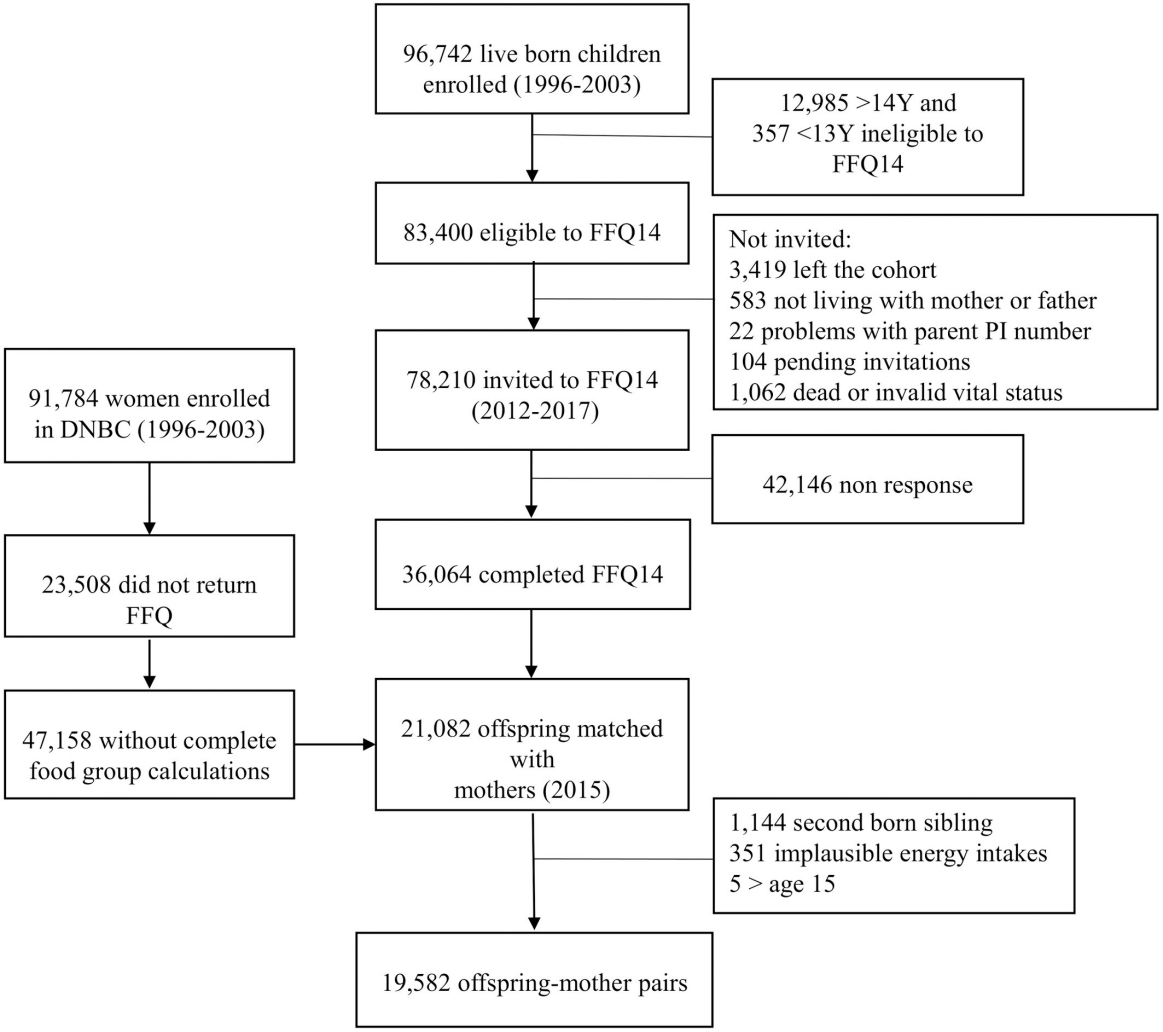
Flowchart. DNBC, Danish National Birth Cohort; FFQ, food frequency questionnaire.

For the present study, offspring with dietary data collected from 2012 until fall 2015 (*n* approximately 24,000) and corresponding mothers (*n* = 68,240) whose maternal pregnancy dietary intake was available were included and matched into 21,082 mother–offspring pairs (Fig 1). No exclusion criteria were set in terms of health status. However, we excluded implausible total energy intakes (<2,500 kJ/day and > 25,000 kJ/day) (*n* = 351), multiple pregnancies and births (*n* = 1,144), and age ≥15 years (*n* = 5) which resulted in a total of 19,582 mother–offspring pairs. Offspring classification of body mass index (BMI) into underweight, normal weight, and overweight/obese was defined according to the International Obesity Task Force (IOTF), which corresponds to adult BMI < 18.5 kg/m^2^, BMI = 18.5–24.9 kg/m^2^, and BMI ≥ 25 kg/m^2^, respectively [17]. This study is reported as per the Strengthening the Reporting of Observational Studies in Epidemiology (STROBE) guidelines (S1 Text). The primary analyses conducted in this study were part of a prospectively written document in form of a PhD protocol performed at The University of Copenhagen. The secondary and supplementary analyses were not prospectively described but were conducted ad hoc and as responses to reviewer comments.

### Assessment of dietary intake

The maternal FFQ completed during pregnancy was based on a questionnaire from the Danish Cancer Registry [18]. The FFQ addressed approximately 360 food items and supplement use during the previous four weeks. Answer options ranged from ‘never’ to ‘8 or more times per week’ [16]. The FFQ was previously validated against 7-day food diaries and urine nitrogen excretion (*n* = 88). The FFQ at age 14 years was developed based on the validated FFQ used in the Growing Up Today Study [19,20] and was completed by the offspring. The offspring FFQ addressed approximately 158 food items, as well as questions about physical activity and supplement use during the previous year. Answer options ranged from ‘did not drink/consume the last year’ to ‘4 times or more per day’. For all food items in the maternal and in the offspring FFQ, standard portion sizes and in-house–developed mixed-dish recipes were applied. Frequency of intake was computed into grams per day using the nutrition software package FoodCalc v.3 [21] combined with the Danish food table [22].

### Assessment of diet quality using a Healthy Eating Index

To evaluate overall diet quality in the study population we applied a newly developed Danish Healthy Eating Index (HEI). The Danish HEI was developed within the DNBC and based on the Danish National Food-Based Dietary Guidelines (FBDG). The FBDG consist of nine guidelines on food intake and one overall guideline including variety, overall food quantity, and physical activity [23]. We adapted seven of the FBDGs and created eight components: fruits and vegetables, fish, dietary fibre, red meat, saturated fatty acids (SFAs), sodium, SSBs, and added sugar. We calculated and evaluated intake in grams per day among offspring and women in relation to the guidelines even though the FBDG guidelines are expressed as an intake of 10 MJ per day. In our study, total energy intake (mean [±standard deviation (SD)]) was 10.7 (2.7) MJ/d and 9.8 (3.7) MJ/d for mothers and offspring, respectively.

The nutrients were retrieved from the maternal and offspring nutrient calculations. Food groups were combined of individual foods available in the FFQs. According to the dietary guidelines, intake of fruits and vegetables, dietary fibres, and fish should be increased to receive positive score (adequate components), whereas intake of the remaining five components (moderation components: red meat, SFAs, sodium, SSBs, and added sugar) should be decreased to receive positive score. Specific cutoff values for the components fruits and vegetables, fish, red meat, dietary fibres, and SSBs were derived from the FBDG. For the nutrients, cutoff values were derived from the Nordic Nutrition Recommendations (NNR) [24]. Proportional scores were assigned in a linear way to intakes between minimum (zero points) and maximum scores (10 points). The eight component scores were summed up to a total score ranging from zero to 80 points. An overview of components and minimum and maximum scores is presented in Table 1.

**Table 1:** The HEI developed within the DNBC.

Components in the DNBC HEI		Danish Dietary Guidelines and Nutrient Recommendations^1^
1 Fruits and vegetables^2^	0 g = 0 points ≥600 g = 10 points	≥600 g per day of fruits and vegetables, where at minimum half is vegetables^3^
2 Dietary fibres	0 g = 0 points ≥30 g = 10 points	≥30 g per day^3^
3 Fish	0 g = 0 points ≥350 g = 10 points	≥350 g fish per week^3^
4 Red meat	>500 g = 0 points ≤200 g = 10 points	≤500 g per week^3^
5 SFAs	≥10E% = 0 points 0E% = 10 points	<10E% per day^4^
6 Sodium	>2.4 g = 0 points ≤1.6 g = 10 points	≤2.4 g sodium per day (4–6 g of salt corresponds to 1.6–2.4 g sodium)^4^
7 SSBs	>500 g = 0 points 0 g = 10 points	≤500 ml per week^3^
8 Added sugar	≥10E% = 0 points 0E% = 10 points	<10% of the total energy intake^4^

**Abbreviations**: DNBC, Danish National Birth Cohort; HEI, Healthy Eating Index; SFA, saturated fatty acid; SSB, sugar-sweetened beverage.

The scoring is continuous for intakes between the minimum and maximum cutoff for intakes.

^1^Expressed as an intake of 10 MJ per day.

^2^If intake of fruits was higher than vegetables, then the score was multiplied by 0.5.

^3^Ministry of Environment and Food of Denmark [23].

^4^Nordic Nutrition Recommendations 2012 [24].

### Covariate assessment

The following variables, retrieved from interviews in GW12 and GW30, were included as covariates: maternal age (continuous and groups: ≤25 years, 26–30 years, 31–35 years, ≥36 years), maternal prepregnancy BMI (underweight BMI < 18.5 kg/m2, normal weight BMI 18.5–24.9 kg/m^2^, overweight BMI 25–29.9 kg/m^2^, obese BMI ≥ 30 kg/m2), parity (0, 1, 2+ children), physical activity during pregnancy (low: <4 h/week, high: ≥4 h/week), alcohol use during pregnancy (yes/no), smoking during pregnancy (never, occasionally, daily), lactation (≤1 m, 2–6 m, ≥7 m), parental educational level (high: more than 4 years of post-secondary education corresponding to a master’s or PhD degree; medium: corresponding to 3–4 years of post-secondary education (corresponding to bachelor’s degree); skilled worker: corresponding to nine years of basic school plus five years of vocational training; and finally unskilled worker). Mother’s age, educational level, BMI, and lactation are known predictors of offspring dietary habits [25–28]. Parity, physical activity, alcohol use, and smoking were included in order to adjust for potential confounding due to social and behavioural factors related to offspring lifestyle.

### Statistical analyses

Participant characteristics were evaluated using descriptive statistics. Deviations from normality for dietary variables were assessed using histograms, and data were presented as median (25th–75th percentile). Categorical variables were presented as frequencies or percentages. The nonparametric Kruskal–Wallis test was applied for skewed variables.

In primary analyses, we divided mother and offspring HEI score into quartiles. We used a log-linear binomial model to estimate risk ratio (RR) and 95% confidence intervals (CIs) for the offspring in the highest quartile of HEI with respect to maternal diet quality as assessed by HEI, with quartile 1 being the reference. Analyses were performed using PROC GENMOD in SAS (SAS 9.4, Cary, NC, USA). Because having a high HEI indicates having a more optimal diet according to dietary guidelines, we denoted relative chance as RR. When testing for linear trend, the quartile variable was coded with the median HEI value in each quartile and entered as a continuous term to calculate *p* for trend. To adjust for potential confounding two models were applied: in model A, RR was adjusted for maternal age, prepregnancy BMI, parity, education, lactation, physical activity level, alcohol use, and smoking during pregnancy. In model B, additional adjustment was made for offspring sex and total energy intake of offspring (quartiles) to account for any potential correlation between offspring HEI score and energy intake. To examine potential sex differences in offspring, we tested for interaction followed by stratified analyses.

Missing values for covariates ranged from 0.02% (maternal age groups) to 4.7% (prepregnancy BMI). Because of the relatively low percentage of missing values (<5%), missing values for maternal age were imputed using the median values, while smoking, physical activity, and parity were imputed using mode. For prepregnancy BMI, which had the highest frequency of missing, missing values were assigned to a separate missing category.

In secondary analyses, a potential effect modification was evaluated in strata of prepregnancy BMI, level of education (proficiency), and smoking during pregnancy. In addition, by including one HEI component at a time into the model, we evaluated if any potential association was driven by specific HEI components. We further examined any potential correlations between the HEI scores of the mother and the offspring. To evaluate potential selection bias due to cohort attrition, we compared maternal characteristics of the offspring who completed the FFQ at age 14 (*n* = 19,582) with those invited who did not complete the FFQ at 14 years of age (*n* = 29,433). One-way ANOVA and chi-square tests were used to test for differences in continuous and categorical characteristics of participants, respectively. To take diseases potentially affecting eating behaviour among women—for instance, anorexia and inflammatory bowel disease, which would result in very low energy intakes and extremely low BMI—into consideration, we did several sensitivity analyses applied in model B: a) excluding women with missing or very low BMI (≤18.5 kg/m^2^, *n* = 517), b) excluding women with low energy intakes (<6.0 MJ/d, *n* = 1,723), and c) excluding vegan/vegetarian mothers (*n* = 249). The HEI score as continuous variable was applied in a sensitivity analyses in the fully adjusted model B.

In a subsample of 595 mother–offspring pairs, the mother’s diet was assessed concurrently with offspring diet 14 years after pregnancy. In this sample, we examined triangular associations between mother’s HEI at pregnancy and mother–offspring HEI at 14-year follow-up. This was done by adjusting the main analysis (model B) for mother’s HEI score assessed 14 years after pregnancy (S1 Appendix).

All tests were two-sided, and we used a threshold of *p* < 0.05 to denote statistical significance. All statistical analyses were performed in the statistical program SAS version 9.4 (SAS Institute).

## Results

### Study participants

The mean age (±SD) among mothers was 30.7 (±4.1) years; the majority of mothers were classified as normal weight (67%) and did not smoke during pregnancy (79%). When dividing the population according to maternal HEI score during pregnancy, there was a significant difference across quartiles of the HEI score for all maternal characteristics. Most of the offspring were classified as normal weight (76%). The prevalence of offspring overweight was slightly higher in the lowest two quartiles of maternal HEI score (Table 2).

**Table 2:** Maternal characteristics (*n* = 19,582).

*Maternal Characteristics*	Overall (23.5 ± 7.4 points)	HEI Score (Mean ± SD)				*p*^1^
		Q1 (14.7 ± 2.6 points)	Q2 (20.6 ± 1.6 points)	Q3 (25.4 ± 1.5 points)	Q4 (33.4 ± 4.5 points)	
Age (years)	30.7 ± 4.1	29.7 ± 3.9	30.6 ± 4.1	30.9 ± 4.0	31.5 ± 4.1	<0.05
Prepregnancy BMI^2^	23.3 ± 3.9	23.8 ± 4.2	23.4 ± 3.9	23.1 ± 3.7	22.7 ± 3.6	<0.05
Underweight, %	4	5	4	4	5	<0.001
Normal weight, %	67	63	66	69	71	
Overweight, %	18	21	20	17	14	
Obese, %	6	8	7	5	5	
Missing, %	5	3	3	4	5	
Physical activity during pregnancy (≥4 h/w), %	9	5	7	8	13	<0.001
Smoking during pregnancy, %	21	26	22	20	17	<0.001
Alcohol in midpregnancy (yes) %	47	45	50	50	45	<0.001
Lactation (≤1 month), %	31	33	30	29	30	<0.001
2–6 months, %	20	25	21	19	15	
≥7 months, %	49	42	49	52	55	
Parous women, %	51	53	53	51	46	<0.001
Parental education, %						<0.001
High	25	19	24	27	31	
Medium	36	34	36	36	38	
Skilled workers	25	31	26	23	18	
Unskilled/students/unemployed	14	17	14	14	13	
*Offspring Characteristics*						
Girls, %	52.6	52.6	51.8	52.7	53.2	0.57
Age (years)	14.0 ± 0.0					
BMI^3^	19.2 ± 2.8	19.4 ± 2.7	19.3 ± 2.7	19.2 ± 2.6	19.0 ± 2.5	<0.001
Underweight, %	15	15	15	14	16	
Normal weight, %	76	75	76	77	77	
Overweight and obese, %	9	10	10	9	7	

^1^*P*-values were evaluated by using the one-way ANOVA for normal distributed continuous covariates, Kruskal–Wallis for non-normal distributed continuous covariates, and chi-square test for categorical covariates.

^2^Underweight BMI < 18.5 kg/m^2^, normal weight BMI 18.5–24.9 kg/m^2^, overweight BMI 25–29.9 kg/m^2^, obese BMI ≥ 30 kg/m^2^.

^3^Child BMI: age- and sex-specific classified by Cole and colleagues [17].

**Abbreviations**: BMI, body mass index; HEI, Healthy Eating Index; Q, quartile; SD, standard deviation.

When we compared maternal characteristics for those offspring who completed the FFQ14 (*n* = 19,582) with those invited who did not complete the FFQ14 (*n* = 29,433) we found that those who completed the FFQ14 had significantly older mothers (mean difference [95% CI]: 0.59 years [0.51, 0.67], *p* < 0.001), mothers had lower prepregnancy BMI (mean difference [95% CI]: −0.49 kg/m^2^ [−0.57, 0.41], *p* < 0.001), and a higher proportion had a higher educational level (25% versus 20%, *p* < 0.001). We observed no significant difference for level of parity between mothers of those offspring who completed the FFQ14 (49% had no prior children) compared with those where the offspring did not complete the FFQ14 (48% had no prior children, *p* = 0.20). The proportion of girls was significantly higher (*p* < 0.001) among adolescents who completed the FFQ14 (52%) compared to those who did not complete the FFQ14 (46%) (S1A Table).

### Maternal and offspring diet quality

The mean HEI score (± SD) for mothers and offspring was 24 points (7) and 24 points (9), respectively (Table 3). For those ranked in quartile 1 of the HEI score, the mean score was 15 points (SD: 3) and 14 points (SD: 3) for mother and offspring, respectively. Corresponding values for those ranked in the highest quartile (quartile 4) were 33 points (SD: 5) and 35 points (SD: 5), respectively.

**Table 3:** Maternal pregnancy and offspring dietary habits according to HEI in Qs (*n* = 19,582).

		***Maternal HEI in Qs***			
**Maternal Dietary Intake**	All	Q1 (*n* = 4,879)	Q2 (*n* = 4,948)	Q3 (*n* = 4,898)	Q4 (*n* = 4,857)
		Mean (± SD) or Median (25th–75th Percentile)			
Mean HEI score^a^	24 (7)	15 (3)	21 (1)	25 (1)	33 (5)
Fruits and vegetables (g/day)^c^	165 (107–219)	113 (74–162)	153 (101–196)	178 (136–227)	218 (166–312)
Dietary fibres (g/day)^b^	28 (10)	23 (8)	27 (8)	30 (9)	32 (10)
Fish (g/week)^c^	159 (92–250)	96 (53–141)	150 (94–214)	197 (120–282)	245 (148–345)
Red meat (g/week)^c^	479 (349–642)	581 (481–720)	519 (413–683)	450 (341–623)	316 (215–444)
E% SFAs^b^	13 (3	14 (3)	13 (3)	12 (3)	11 (3)
Sodium (mg/day)^b^	3.3 (0.9)	3.2 (0.7)	3.4 (0.9)	3.5 (1.0)	3.3 (1.0)
SSBs (l/week)^c^	1.51 (0.82–2.56)	2.00 (1.21–3.33)	1.61 (0.93–2.61)	1.38 (0.79–2.35)	1.05 (0.47–1.99)
E% added sugar ^c^	7 (5–10)	10 (7–13)	7 (5–10)	6 (4–8)	5 (4–7)
Energy (MJ/day)^b^	10.6 (2.7)	10.4 (2.4)	10.8 (2.6)	10.8 (2.8)	10.3 (2.9)
Protein (E%)^b^	15 (2)	15 (2)	15 (2)	15 (2)	16 (2)
Fat (E%)^b^	31 (6)	33 (5	32 (5)	30 (6)	28 (6)
Carbohydrate (E%)^b^	54 (6)	53 (5)	53 (5)	54 (6)	56 (6)
		***Offspring HEI in Qs***			
**Offspring Dietary Intake**	All	Q1 (*n* = 4,862)	Q2 (*n* = 4,886)	Q3 (*n* = 4,924)	Q4 (*n* = 4,910)
		Mean (± SD) or Median (25th–75th Percentile)			
Mean HEI score^a^	24 (9)	14 (3)	20 (2)	26 (2)	35 (5)
Fruits and vegetables (g/day)^c^	279 (169–435)	172 (113–243)	266 (176–366)	346 (214–490)	440 (274–625)
Dietary fibres (g/day)^c^	26 (19–36)	20 (16–25)	27 (20–34)	30 (21–40)	33 (23–46)
Fish (g/week)^c^	67 (30–120)	43 (16–75)	64 (30–106)	81 (41–131)	103 (50–217)
Red meat (g/week)^c^	666 (463–930)	699 (550–917)	705 (507–958)	677 (450–957)	531 (450–869)
E% SFAs^b^	12 (2)	13 (2)	12 (2)	11 (2)	10 (2)
Sodium (mg/day)^b^	3.3 (1.2)	3.0 (0.9)	3.3 (1.3)	3.4 (1.5)	3.2 (1.7)
SSBs (l/week)^c^	0.63 (0.31–1.38)	1.25 (0.68–1.95)	0.76 (0.47–1.56)	0.56 (0.30–1.02)	0.27 (0.13–0.48)
E% added sugar^c^	6 (4–9)	10 (8–12)	7 (5–9)	6 (4–7)	4 (3–6)
Energy (kJ/day)^b^	9.9 (3.7)	9.1 (2.7)	10.0 (3.5)	10.2 (3.9)	10.1 (4.5)
Protein (E%)^b^	16 (2)	15 (2)	15 (2)	16 (2)	16 (2)
Fat (E%)^b^	32 (5)	33 (4)	33 (4)	32 (4)	30 (5)
Carbohydrate (E%)^b^	52 (5)	50 (5)	51 (5)	52 (5)	53 (6)

^a^The total score of eight components: fruits and vegetables, fish, dietary fibres, red meat, SFAs, sodium, SSBs, and added sugar. The score can range from zero to 80.

^b^One-way ANOVA test significant for normally distributed variables, *p* <0.001

^c^ Kruskal–Wallis test significant for non-normally distributed variables, *p* < 0.001.

**Abbreviations**: HEI, Healthy Eating Index; Q, quartile; SD, standard deviation; SFA, saturated fatty acid; SSB, sugar-sweetened beverage.

As expected, maternal and offspring intake of fruits and vegetables, dietary fibre, and fish increased with increasing HEI scores, while the opposite was observed for red meat (mothers only), SFAs, SSBs, and added sugars (Table 3). However, no trend was observed for sodium and total energy intake across the HEI quartiles. For sodium and energy intake, we additionally observed essentially no correlation between offspring intake and offspring HEI score (*r* = 0.004 and 0.06, respectively) (Table 4). The maternal HEI score was correlated positively with offspring HEI score (Pearson *r* = 0.22, *p* < 0.001).

**Table 4:** Correlation coefficients between the HEI and offspring dietary intake.

Offspring Dietary Habits (*n* = 19,582)	Correlation Coefficients*	
	Offspring HEI Score	Mothers’ HEI Score
HEI score		0.22
Fruits and vegetables	0.52	0.48
Dietary fibres	0.38	0.37^a^
Fish	0.34	0.48
Red meat	−0.19	−0.48
SFAs	−0.47^a^	−0.35^a^
Sodium	0.004^a^	0.03^a^
SSBs	−0.55	−0.28
Added sugar	−0.64	−0.50
Energy	0.06^a^	−0.02^a^

^a^Pearson correlations; otherwise Spearman correlations,

*p < 0.001 for all correlations tested on maternal and offspring HEI as continuous score.

**Abbreviations**: HEI, Healthy Eating Index; SFA, saturated fatty acid; SSB, sugar-sweetened beverage.

### Association between maternal and offspring diet quality

Main analyses are presented in Table 5 showing the RR of the offspring in the highest HEI quartile at 14 years of age across quartiles of maternal HEI scores during pregnancy. The RR of the offspring with a high HEI score was 2-fold increased when comparing the highest to the lowest maternal quartile (RR: 2.1; 95% CI: 1.9, 2.3). After adjustment for maternal characteristics (model A) and additionally offspring sex and total energy intake (model B), the RR estimates were only slightly attenuated and remained significant (RR: 1.9; 95% CI: 1.8, 2.1).

**Table 5:** Maternal prenatal dietary habits as predictor of offspring dietary habits at age 14 (*n* = 19,582).

Maternal HEI Q	Cases^a^/N	RR	RR Model A^b^	RR Model B^c^
Q1	812/4,879	1	1	1
Q2	1,080/4,948	1.31 (1.21, 1.42)	1.28 (1.18, 1.39)	1.29 (1.18, 1.42)
Q3	1,292/4,898	1.58 (1.47, 1.71)	1.52 (1.40, 1.64)	1.53 (1.40, 1.67)
Q4	1,726/4,857	2.14 (1.98, 2.30)	1.98 (1.83, 2.13)	1.97 (1.81, 2.15)
*p* for trend		<0.001	<0.001	<0.001

Dietary habits are quantified in terms of adherence to dietary recommendations estimated by the HEI, and the RR of the offspring being in the highest Q is modelled. **Abbreviations**: BMI, body mass index; HEI, Healthy Eating Index; Q, quartile; RR, risk ratio.

^a^Cases = the number of offspring in Q4 according to ranking of the mother.

^b^Adjusted for maternal age, parity, education, physical activity, smoking and alcohol intake during pregnancy, and lactation.

^c^Additionally adjusted for offspring energy intake and sex.

In Table 5, results were presented based on categorising offspring and maternal HEI into quartiles. When examining the association between maternal–offspring HEI on a continuous scale, a per 10-unit increase of maternal HEI score during pregnancy was associated with 2.3 (95% CI: 2.1, 2.4) unit increase in offspring HEI score 14 years later. The maternal HEI score explained around 14% of the variance in the offspring HEI score.

Despite an almost 2-fold increase in RR for the offspring being in the highest HEI quartile if the mother was in the highest quartile during pregnancy, the variation in offspring HEI within each maternal quartile was substantial and overlapping with adjacent quartiles (S1 Fig). We observed no change in direction or magnitude in the sex-stratified, fully adjusted analyses (model B) for boys (RR: 1.9; 95% CI: 1.7, 2.3) or girls (RR: 1.9; 95% CI: 1.8, 2.2), respectively (S2A Table).

In secondary analyses, we examined further potential modification by maternal characteristics in stratified analyses by maternal prepregnancy BMI (Table 6), parental education, and smoking during pregnancy (S2B Table). RR estimates were comparable to and in the same direction as those from the nonstratified analyses (as presented in Table 5). Furthermore, to evaluate whether one HEI component was responsible for the association between maternal–offspring diet, we added one single component of maternal HEI at a time to model B. The results were within the same direction as the results presented in Table 5, and no specific component appeared to be responsible for the relationship between maternal and offspring HEI scores (S3 Table).

**Table 6:** Maternal prenatal dietary habits as predictor of offspring dietary habits; BMI strata.

Maternal HEI Q	Cases^a^/N	RR	RR Model A^b^	RR Model B^c^
*Prepregnancy BMI*^d^ *(kg/m*^*2*^*)*				
Underweight	802			
Q1	27/178	1.00	1.00	1.00
Q2	34/191	1.17 (0.74, 1.86)	1.16 (0.73, 1.84)	1.11 (0.75, 1.63)
Q3	56/205	1.80 (1.19, 2.72)	1.76 (1.16, 2.66)	1.54 (1.08, 2.20)
Q4	93/228	2.69 (1.84, 3.93)	2.51 (1.69, 3.72)	2.05 (1.47, 2.86)
*p* for trend		<0.001	<0.001	<0.001
Normal weight	13,170			
Q1	500/3,070	1.00	1.00	1.00
Q2	720/3,249	1.36 (1.23, 1.50)	1.32 (1.19, 1.46)	1.33 (1.20, 1.47)
Q3	903/3,380	1.64 (1.49, 1.80)	1.56 (1.41, 1.72)	1.54 (1.40, 1.70)
Q4	1,232/3,471	2.17 (1.99, 2.39)	1.99 (1.81, 2.19)	1.96 (1.78, 2.15)
*p* for trend		<0.001	<0.001	<0.001
Overweight	3,464			
Q1	192/1,003	1.00	1.00	1.00
Q2	213/972	1.14 (0.96, 1.36)	1.12 (0.94, 1.33)	1.13 (0.95, 1.34)
Q3	215/828	1.36 (1.14, 1.61)	1.30 (1.10, 1.55)	1.32 (1.11, 1.56)
Q4	223/661	1.76 (1.49, 2.08)	1.67 (1.41, 1.98)	1.66 (1.41, 1.96)
*p* for trend		<0.001	<0.001	<0.001
Obese	1,225			
Q1	61/404	1.00	1.00	1.00
Q2	68/326	1.38 (1.01, 1.89)	1.38 (1.01, 1.89)	1.35 (0.99, 1.83)
Q3	59/265	1.47 (1.07, 2.04)	1.49 (1.08, 2.06)	1.48 (1.07, 2.03)
Q4	78/230	2.25 (1.68, 3.01)	2.26 (1.68, 3.04)	2.15 (1.61, 2.87)
*p* for trend		0.001	<0.001	<0.001

^a^Cases = the number of offspring in Q4 according to ranking of the mother.

^b^Adjusted for maternal age, parity, education, physical activity, smoking and alcohol intake during pregnancy, and lactation.

^c^Additionally adjusted for offspring energy intake and sex.

^d^Underweight <18.5 kg/m^2^, normal weight 18.5–24.9 kg/m^2^, overweight 25–29.9 kg/m^2^, obese ≥ 30 kg/m^2^.

**Abbreviations**: BMI, body mass index; HEI, Healthy Eating Index; Q, quartile.

In sensitivity analyses, we examined potential diseases or conditions that could affect dietary intake in mothers. Exclusion of mothers with very low energy intakes (<6.0 MJ/d), mothers with low prepregnancy BMI (≤18.5 kg/m^2^), and mothers who claimed to be a vegetarian during pregnancy were used as proxy for anorexia or inflammatory bowel disease. The exclusion of these women resulted in slightly decreased risk estimates; however, they remained significant and in the same direction (S1B Table).

A description of and results from analyses in the subsample for the triangular associations between mother–offspring HEI at pregnancy and follow-up is shown in S1A Appendix Text. When comparing mothers in the subsample with mothers in the main study, minor differences were observed for smoking during pregnancy and parity (S1B Appendix Table). No differences were observed for number of girls or categorisation of BMI (S1C Appendix Table). In the subsample, we observed relatively low (*r* < 0.4) significant correlations for all associations examined (*r* from 0.25 to 0.37) (S1D Appendix Fig). The HEI score of mothers in the subsample was significantly higher (*p* < 0.001) compared with the full study population (mean HEI score [SD]: 26 [8] points), whereas the HEI score among the subsample offspring was within the same magnitude as the full offspring study population (23 [9] points). Adjustment for maternal HEI score assessed at 14 years after pregnancy had limited impact on the association between maternal diet quality in pregnancy and offspring diet quality at 14 years. The RR of 1.8 (95%CL: 1.0, 3.1) was a similar estimate as the one obtained for the full sample in Table 5 (S1E Appendix Table).

## Discussion

In this study of approximately 20,000 mother–child pairs, we found that offspring’s diet quality was positively associated to their mother’s antenatal diet quality, despite the fact that the two dietary assessments were undertaken 15 years apart. This association was observed at a time when offspring become increasingly independent and rely more on own food preferences, and it was robust after adjustment for a number of different sociodemographic factors and consistently observed across strata of offspring sex, maternal BMI, and education status.

Several different mechanisms could be underlying the observed associations. They could be driven by biological factors, social determinants, or both. Results from randomised controlled trials support the hypothesis that maternal dietary intake during pregnancy influences later dietary intake of the offspring [29,30]. However, women with a relatively healthy diet during pregnancy are also likely to continue such habits postnatally [14], which will to some extent be reflected in offspring dietary habits at a young age. This may explain the relatively strong correlation observed in the study by Asham and colleagues in which follow-up was conducted 2–3 years after birth. In that study, the correlation coefficient between diet quality in pregnancy and postnatally was 0.85, while the cross-sectional correlation between maternal–offspring diet quality was 0.66 [9]. These correlations are considerably stronger than those (*r* approximately 0.3) observed in our study at the 14-year follow-up and those observed in other cross-sectional studies between parents and 8- to 12-year–old children (*r* approximately 0.2–0.3) [31, 32].

If mothers in our cohort had continued the dietary pattern similar to the one estimated during pregnancy, we might have seen a decreased risk estimate when adjusting for maternal characteristics reflecting healthier antenatal lifestyles, but no such decrease was observed. Furthermore, in a subset of our participants, maternal diet quality assessed concurrently with offspring diet quality 14 years after pregnancy did not seem to confound the observed association. In contrast to findings by Asham and colleagues, in which the prenatal–offspring association seemed to be mediated by postnatal dietary intake, these results point to a relatively modest influence of mother’s current diet quality on 14-year–old offspring diet quality.

Other factors that may influence dietary habits in childhood and adolescence include attitudes towards healthy eating, familial shared behaviours, peer pressure, and social acceptability [26, 33]. These factors will have more influence on later childhood and adolescence, resulting in greater variation of dietary habits, and thus may, to some extent, explain the modest correlations and substantial variation in offspring HEI observed in this age group.

Our study has considerable strengths. Because of the large sample size and detailed covariate assessment, we were able to explore in detail the stability of our findings across strata of maternal characteristics that could confound or modify the association between mother and offspring diet. In addition, the long follow-up in our study is unique because it allows us to explore the influence of maternal diet on offspring’s own dietary habits at a time when one would expect that the offspring’s start making own food choices. Dietary intake among women was assessed by a detailed FFQ validated against 7-day weighted food records during pregnancy [34], and a detailed FFQ validated against 3 × 24-h recalls was applied to collect dietary data among offspring [35]; in the validation analyses, both the maternal and offspring FFQ showed moderate Spearman correlations for energy intake of *r* = 0.42 and *r* = 0.54, respectively. A number of limitations within the study are acknowledged. The FFQ method has some limitations because it relies on the participant’s ability to recall and report dietary intake, and calculation relies on the content of the applied food table. If memory bias affected the accuracy of assessed dietary intake equally among participants, level of diet quality might have been skewed in either direction compared with true intake, which would not affect ranking across groups. A registry-based study conducted at the time found no indication of attrition bias with respect to maternal smoking and adverse pregnancy outcomes such as preterm delivery and low birth weight (<2,500 g) [36]. However, as with all longitudinal studies with long-term follow-up, there is substantial attrition. In our 20-year follow-up, approximately 50% of those invited agreed to participate, which is not unexpected among young adults. The analysis on participants versus nonparticipants in the FFQ14 follow-up study indicated some level of attrition bias towards healthier participants and higher socioeconomic status. The implication is that those with poorer diet quality might be underrepresented in our study population, but at the same time, it is not clear how such bias could lead to spurious correlation between maternal–offspring dietary habits. We did not have the possibility to adjust for the potential confounding of, for instance, unhealthy habits such as smoking and socioeconomic status of the mother, which could have changed systematically after pregnancy. Confounding by later environmental exposures could occur if these were somehow related to changes in dietary habits. However, we would still think it is more likely that socioeconomic and healthy lifestyle habits would remain fairly constant for most subjects. In any case, the positive association we observe is more in the direction that one would expect rather than the opposite.

The HEI applied in our study summarised diet quality in mothers and offspring relative to current Danish dietary guidelines. Even though the HEI score for the mothers was based on current dietary guidelines that did not apply 10–15 years ago, it did nevertheless give an estimate of women’s diet quality during pregnancy that could be compared with that of her child’s. The level of intake assessed with the maternal and offspring FFQs, respectively, was comparable with the level assessed by food records in a similar age groups in the Danish National Surveys [37, 38].

Irrespective of which mechanisms may be underlying the observed associations between characteristics of the mother’s diet in pregnancy and the offspring’s diet many years later, our findings have relevant implications for studies focusing on environmental factors operating during the years of development on aetiologies of adult diseases. Our results emphasise the need for taking offspring diet quality into account when studying the association between dietary factors in pregnancy and offspring adult health, and—vice versa—the need for taking dietary factors in pregnancy into account when studying associations between dietary exposures in adolescent years and adult health.

### Conclusion

In conclusion, the maternal diet quality during pregnancy was associated with diet quality of the offspring at age 14 years. These findings indicate the importance of separating early dietary exposures from later dietary exposures when studying dietary aetiologies of diseases postulated to have developmental origins such as, for instance, obesity or asthma, in observational settings.

## Supporting information

S1 Text STROBE guideline (pdf format). STROBE, Strengthening the Reporting of Observational Studies in Epidemiology.
(PDF)

S1 Table ‘Analyses of attrition’ includes A: A comparison of maternal characteristics for those offspring who completed the FFQ14 and those invited who did not complete the FFQ14 and B: Results from analyses in which specific groups of women with characteristics that may affect eating behaviour are excluded (pdf format). FFQ, food frequency questionnaire.
(PDF)

S2 Table ‘Stratified analyses’ (pdf format) includes A: Results of maternal prenatal dietary habits as predictor of 14-year–old offspring dietary habits stratified by boys and girls and B: Results of maternal prenatal dietary habits as predictor of 14-year–old offspring dietary habits stratified by maternal education and smoking during pregnancy. (PDF)

S3 Table ‘Adjustment for the individual maternal HEI components’ (pdf format). HEI, Healthy Eating Index.
(PDF)

S1 Fig The figure illustrates the relationship between the distribution in offspring HEI score and maternal HEI score in quartiles (pdf format). HEI, Healthy Eating Index.
(PDF)

S1 Appendix ‘Subsample analyses’ (pdf. format) includes A Text: Background, methods, and results from triangular association analyses between mother–offspring HEI at pregnancy and at 14 years follow-up; B Table: ‘maternal characteristics of subsample compared with the main study’; C Table: ‘offspring characteristics of subsample compared with the main study’; D Figure: ‘Spearman correlations coefficients in subsample analyses’; E Table: ‘results from triangular association analyses between mother–offspring HEI at pregnancy and at 14 years follow-up’. HEI, Healthy Eating Index.
(PDF)

## Abbreviations

BMI: body mass index
CI: confidence interval
DNBC: Danish National Birth Cohort
DOHaD: Developmental Origins of Health and Disease
FBDG: Food-Based Dietary Guidelines
FFQ: food frequency questionnaire
GW: gestation week
HEI: Healthy Eating Index
IOTF: International Obesity Task Force
NNR: Nordic Nutrition Recommendations
RR: risk ratio
SD: standard deviation
SFA: saturated fatty acid
SSB: sugar-sweetened beverage
STROBE: Strengthening the Reporting of Observational Studies in Epidemiology

## References

[1] Hansen S, Strom M, Maslova E, Dahl R, Hoffmann HJ, Rytter D, et al Fish oil supplementation during pregnancy and allergic respiratory disease in the adult offspring. J Allergy Clin Immunol. 2017;139(1):104–11.e4. 10.1016/j.jaci.2016.02.042 27246522

[2] Hrolfsdottir L, Halldorsson TI, Rytter D, Bech BH, Birgisdottir BE, Gunnarsdottir I, et al Maternal Macronutrient Intake and Offspring Blood Pressure 20 Years Later. J Am Heart Assoc. 2017;6(4): e005808 10.1161/JAHA.117.005808 28438741 PMC5533044

[3] Boney CM, Verma A, Tucker R, Vohr BR. Metabolic syndrome in childhood: association with birth weight, maternal obesity, and gestational diabetes mellitus. Pediatrics. 2005;115(3):e290–6. 10.1542/peds.2004-1808 15741354

[4] Clausen TD, Mathiesen ER, Hansen T, Pedersen O, Jensen DM, Lauenborg J, et al High prevalence of type 2 diabetes and pre-diabetes in adult offspring of women with gestational diabetes mellitus or type 1 diabetes: the role of intrauterine hyperglycemia. Diabetes Care. 2008;31(2):340–6. 10.2337/dc07-1596 18000174

[5] Farvid MS, Eliassen AH, Cho E, Liao X, Chen WY, Willett WC. Dietary Fiber Intake in Young Adults and Breast Cancer Risk. Pediatrics. 2016;137(3):e20151226 10.1542/peds.2015-1226 26908709 PMC4771124

[6] Malik VS, Fung TT, van Dam RM, Rimm EB, Rosner B, Hu FB. Dietary patterns during adolescence and risk of type 2 diabetes in middle-aged women. Diabetes Care. 2012;35(1):12–8. 10.2337/dc11-0386 22074723 PMC3241320

[7] Krupp D, Shi L, Egert S, Wudy SA, Remer T. Prospective relevance of fruit and vegetable consumption and salt intake during adolescence for blood pressure in young adulthood. Eur J Nutr. 2015;54(8):1269–79. 10.1007/s00394-014-0804-y 25410750

[8] Hu FB. Resolved: there is sufficient scientific evidence that decreasing sugar-sweetened beverage consumption will reduce the prevalence of obesity and obesity-related diseases. Obes Rev. 2013;14(8):606–19. 10.1111/obr.12040 23763695 PMC5325726

[9] Sabinsky MS, Toft U, Andersen KK, Tetens I. Development and validation of a Meal Index of dietary Quality (Meal IQ) to assess the dietary quality of school lunches. Public Health Nutr. 2012;15(11): 2091–2099. 10.1017/S1368980012001012 22717318 PMC10271702

[10] Vyncke K, Cruz Fernandez E, Fajo-Pascual M, Cuenca-Garcia M, De Keyzer W, Gonzalez-Gross M, et al Validation of the Diet Quality Index for Adolescents by comparison with biomarkers, nutrient and food intakes: the HELENA study. The British journal of nutrition. 2013;109(11):2067–78. 10.1017/S000711451200414X 23110799

[11] Marshall S, Watson J, Burrows T, Guest M, Collins CE. The development and evaluation of the Australian child and adolescent recommended food score: a cross-sectional study. Nutrition journal. 2012;11:96 10.1186/1475-2891-11-96 23164095 PMC3546018

[12] Chiplonkar SA, Tupe R. Development of a diet quality index with special reference to micronutrient adequacy for adolescent girls consuming a lacto-vegetarian diet. J Am Diet Assoc. 2010 8;110(8):1256. 10.1016/j.jada.2010.03.016 20497784

[13] Handeland K, Kjellevold M, Wik Markhus M, Eide Graff I, Froyland L, Lie O, et al A Diet Score Assessing Norwegian Adolescents’ Adherence to Dietary Recommendations-Development and Test-Retest Reproducibility of the Score. Nutrients. 2016;8(8): E467 10.3390/nu8080467 27483312 PMC4997380

[14] Ashman AM, Collins CE, Hure AJ, Jensen M, Oldmeadow C. Maternal diet during early childhood, but not pregnancy, predicts diet quality and fruit and vegetable acceptance in offspring. Maternal & child nutrition. 2016;12(3):579–90. 25294406 10.1111/mcn.12151 PMC6860109

[15] Olsen J, Melbye M, Olsen SF, Sorensen TI, Aaby P, Andersen AM, et al The Danish National Birth Cohort—its background, structure and aim. ScandJPublic Health. 2001;29(4):300–7. 10.1177/14034948010290040201 11775787

[16] Olsen SF, Mikkelsen TB, Knudsen VK, Orozova-Bekkevold I, Halldorsson TI, Strom M, et al Data collected on maternal dietary exposures in the Danish National Birth Cohort. Paediatr. Perinat. Epidemiol. 2007;21(1):76–86. 10.1111/j.1365-3016.2007.00777.x 17239183

[17] Cole TJ, Bellizzi MC, Flegal KM, Dietz WH. Establishing a standard definition for child overweight and obesity worldwide: international survey. BMJ. 2000;320(7244):1240–3. 10.1136/bmj.320.7244.1240 10797032 PMC27365

[18] Tjønneland AT, Overvad KIM, HaraldsdÓTtir J, Bang S, Ewertz M, Jensen OLEM. Validation of a Semiquantitative Food Frequency Questionnaire Developed in Denmark. International journal of epidemiology. 1991;20(4):906–12. 10.1093/ije/20.4.906 1800429

[19] Rockett HR, Breitenbach M, Frazier AL, Witschi J, Wolf AM, Field AE, et al Validation of a youth/adolescent food frequency questionnaire. PrevMed. 1997;26(6):808–16. 10.1006/pmed.1997.0200 9388792

[20] GUTS, The Growing Up Today Study. [Cited 3 March 2019] [Internet]. http://nhs2survey.org/gutswordpress/.

[21] Lauritsen J. Foodcalc v.1.3. [Cited 3 May 2015] [internet]. github.com/jesperldk/FoodCalc.

[22] Danish Food Composition Data version 7. [Cited 3 May 2015] [Internet]. https://frida.fooddata.dk.

[23] Ministry of Environment and Food of Denmark. De officielle kostråd (Officiel Danish dietary guidelines). [Cited 10 May 2017] [internet]. http://altomkost.dk/deofficielleanbefalingertilensundlivsstil/de-officielle-kostraad/.

[24] The Nordic Nutrition Recommendations 2016. Food & Nutrition Research. 2016;60(1):31961. 27317923 10.3402/fnr.v60.31961 PMC4981751

[25] Pei Z, Flexeder C, Fuertes E, Standl M, Berdel D, von Berg A, et al Mother’s body mass index and food intake in school-aged children: results of the GINIplus and the LISAplus studies. Eur J Clin Nutr. 2014 8;68(8):898–906. 10.1038/ejcn.2014.92 Epub 2014 May 21 24848629 PMC4283383

[26] Finger JD, Varnaccia G, Tylleskar T, Lampert T, Mensink GB. Dietary behaviour and parental socioeconomic position among adolescents: the German Health Interview and Examination Survey for Children and Adolescents 2003–2006 (KiGGS). BMC Public Health. 2015;15:498 10.1186/s12889-015-1830-2 25985772 PMC4492169

[27] Mennella JA. Ontogeny of taste preferences: basic biology and implications for health. Am J Clin Nutr. 2014 3;99(3):704S–11S. 10.3945/ajcn.113.067694 Epub 2014 Jan 22. 24452237 PMC3927698

[28] Andersen LB, Pipper CB, Trolle E, Bro R, Larnkjaer A, Carlsen EM, et al Maternal obesity and offspring dietary patterns at 9 months of age. European journal of clinical nutrition. 2015;69(6):668–75. 10.1038/ejcn.2014.258 25469467

[29] Mennella JA, Jagnow CP, Beauchamp GK. Prenatal and postnatal flavor learning by human infants. Pediatrics. 2001;107(6):E88 10.1542/peds.107.6.e88 11389286 PMC1351272

[30] Hausner H, Nicklaus S, Issanchou S, Molgaard C, Moller P. Breastfeeding facilitates acceptance of a novel dietary flavour compound. Clin Nutr. 2010;29(1):141–8. 10.1016/j.clnu.2009.11.007 19962799

[31] Robinson LN, Rollo ME, Watson J, Burrows TL, Collins CE. Relationships between dietary intakes of children and their parents: a cross-sectional, secondary analysis of families participating in the Family Diet Quality Study. Journal of human nutrition and dietetics: the official journal of the British Dietetic Association. 2015;28(5):443–51. 25130863 10.1111/jhn.12261

[32] Bere E, Klepp KI. Correlates of fruit and vegetable intake among Norwegian schoolchildren: parental and self-reports. Public health nutrition. 2004;7(8):991–8. 10.1079/PHN2004619 15548337

[33] Story M, Kaphingst KM, Robinson-O’Brien R, Glanz K. Creating healthy food and eating environments: policy and environmental approaches. Annu Rev Public Health. 2008;29:253–72. 10.1146/annurev.publhealth.29.020907.090926 18031223

[34] Mikkelsen TB, Olsen SF, Rasmussen SE, Osler M. Relative validity of fruit and vegetable intake estimated by the food frequency questionnaire used in the Danish National Birth Cohort. Scand J Public Health. 2007;35(2):172–9. 10.1080/14034940600975625 17454921

[35] Bjerregaard AA, Halldorsson TI, Kampmann FB, Olsen SF, Tetens I. Relative validity of a web-based food frequency questionnaire for Danish adolescents. Nutrition journal. 2018;17(1):9 10.1186/s12937-018-0312-7 29329542 PMC5767066

[36] Aagaard Nohr E, Frydenberg M, Henriksen Tine, Olsen J. Does Low Participation in Cohort Studies Induce Bias? *Epidemiology* (Cambridge, Mass.). 2006;17:413–8. 10.1097/01.ede.0000220549.14177.60 16755269

[37] Fagt S, Matthiessen J, Trolle E, Lyhne N, Christensen T, Hinsch H-J et al. 2. Danskernes kostvaner 2000–2001. Udviklingen i danskernes kost—forbrug, indkøb og vaner. Søborg, Danmark: Fødevaredirektoratet; 2002. [Cited 13 August 2019] [internet]. Report in Danish https://www.food.dtu.dk/publikationer/ernaering-og-kostvaner/de_nationale_kostundersoegelser

[38] Pedersen AN, T. Christensen, J. Matthiessen, V.K. Knudsen, M. Rosenlund-Sørensen, A. Biltoft-Jensen, HJ. Hinsch, K.Y. Ygil, K. Kørup, E. Saxholt, E. Trolle, A.B. Søndergaard, S.Fagt. Dietary Habits in Denmark 2011–2013. Main results. Report. Søborg, Denmark: National Food Institute; 2015.

