# Correction: Mother’s dietary quality during pregnancy and offspring’s dietary quality in adolescence: Follow-up from a national birth cohort study of 19,582 mother–offspring pairs

**DOI:** 10.1371/journal.pmed.1003004

**Published:** 2019-11-26

**Authors:** Anne Ahrendt Bjerregaard, Thorhallur Ingi Halldorsson, Inge Tetens, Sjurdur Frodi Olsen

The first and fourth authors’ names are published incorrectly in the article XML. The correct name for the first author is Anne Ahrendt Bjerregaard. The correct indexing in PubMed should read Bjerregaard AA. The correct name for the fourth author is Sjurdur Frodi Olsen. The correct indexing in PubMed should read Olsen SF.

The correct citation, therefore, should read: Bjerregaard AA, Halldorsson TI, Tetens I, Olsen SF (2019) Mother’s dietary quality during pregnancy and offspring’s dietary quality in adolescence: Follow-up from a national birth cohort study of 19,582 mother–offspring pairs. PLoS Med 16(9): e1002911. https://doi.org/10.1371/journal.pmed.1002911
