# Supplementary material for: Mother’s dietary quality during pregnancy and offspring’s dietary quality in adolescence: Follow-up from a national birth cohort study of 19,582 mother–offspring pairs
Source: PLoS Med. 2019 Sep 12;16(9):e1002911. doi: 10.1371/journal.pmed.1002911 (PMC6742222; doi:10.1371/journal.pmed.1002911)
Supplement: S1 Text — STROBE, Strengthening the Reporting of Observational Studies in Epidemiology. (PDF) [file pmed.1002911.s001.pdf]

Article title: Mother's dietary quality during pregnancy and offspring's dietary quality in adolescence: follow-up from a nationwide birth cohort study of 19,582 mother-offspring pairs

Author names: Anne Ahrendt Bjerregaard, Thorhallur Ingi Halldorsson, Inge Tetens, Sjurdur Frodi Olsen

Affiliation and e-mail address of corresponding author
Center for Fetal Programming, Department of Epidemiology Research, Statens Serum Institut, Copenhagen, Denmark, anne@ssi.dk

---

STROBE Statement—Checklist of items that should be included in reports of ***cohort studies***

	Item No	Recommendation
**Title and abstract**	1	(*a*) Indicate the study's design with a commonly used term in the title or the abstract<br>*Title: Mother's dietary quality during pregnancy and offspring's dietary quality in adolescence: follow-up from a nationwide birth cohort of 19,582 mother-offspring pairs*
		(*b*) Provide in the abstract an informative and balanced summary of what was done and what was found:<br>*Abstract:*<br>*From 1996 to 2003, the Danish National Birth Cohort (DNBC) was established. Women from across the country were enrolled and dietary intake in mid-pregnancy was assessed with a 360-items food frequency questionnaire. During 2013-2018, dietary intake was assessed concurrently at age 14 years with a 150-items food frequency questionnaire (FFQ) in the DNBC children. For both questionnaires, a Healthy Eating Index (HEI) was developed as an indicator for diet quality.*<br>*The HEI scores was divided into quartiles; individuals in the highest quartile represented those with the most optimal diet. A log-linear binomial model was used to estimate the relative risk of the offspring being in the highest quartile of HEI (Q4) at age 14 years if the mother was ranked in Q4 during pregnancy.*<br>*We found that offspring born to mothers who were in the highest HEI quartile during pregnancy, were more likely themselves to be located in the highest HEI quartile at age 14 years (risk ratio: 2.1, 95% confidence interval: 2.0, 2.3, p < 0.001).*
**Introduction**		
Background/rationale	2	Explain the scientific background and rationale for the investigation being reported<br>*Introduction: Evidence from the field of Developmental Origins of Health and Disease (DOHaD) research has consolidated the hypothesis that certain dietary exposures during foetal life – i.e., certain components of the mother's diet during pregnancy – can have a long-lasting impact on the child's susceptibility to non-communicable diseases*<br>*Studying such relationships represents a significant challenge since they require many years of follow-up, leaving room for multiple factors to potentially contribute in a process that may eventually lead to*

		disease. One such factor is the offspring's own dietary habits.<br>*A potential association between mother's dietary habits during pregnancy and offspring's dietary intake could exist, ultimately influencing later disease risk.*
Objectives	3	State specific objectives, including any prespecified hypotheses<br>*Introduction: The aim of the present study was to elucidate to what extent maternal diet quality during pregnancy is associated with diet quality in the offspring at age 14 years in the DNBC.*
**Methods**		
Study design	4	Present key elements of study design early in the paper<br>*Introduction: The Danish National Birth Cohort (DNBC) is, to our knowledge, one of the first large prospective studies to comprehensively assess dietary intake in pregnancy and again during the adolescent years.*
Setting	5	Describe the setting, locations, and relevant dates, including periods of recruitment, exposure, follow-up, and data collection<br>*Methods: The DNBC was established between 1996 and 2003 and includes ~90 000 offspring from across the country. A follow-up among children at age ~14 years (between 2012 and 2018, n = 36,064) focused on overall dietary intake using a comprehensive web-based FFQ.*<br>*For the present study, offspring with dietary data collected from 2012 until fall 2015 (n ~ 24,000) and corresponding mothers (n = 68,240) whose maternal pregnancy dietary intake was available were included and matched into 21,082 mother-offspring pairs.*
Participants	6	(*a*) Give the eligibility criteria, and the sources and methods of selection of participants. Describe methods of follow-up<br>*Methods: Women who could do interviews in Danish and completed an FFQ in mid-pregnancy (gestation week [GW] 25), were eligible for enrolment in the DNBC.*<br>*No exclusion criteria were set in terms of health status for the present study.*
		(*b*) For matched studies, give matching criteria and number of exposed and unexposed<br>*NA.*
Variables	7	Clearly define all outcomes, exposures, predictors, potential confounders, and effect modifiers. Give diagnostic criteria, if applicable<br>*Methods: Exposure and outcome:*<br>*To evaluate overall diet quality in the study population we applied a newly developed Danish Healthy Eating Index (HEI).*<br>*Page 10, Methods: The nutrients were retrieved from the maternal and offspring nutrient calculations. Food groups were combined of individual foods available in the FFQs. According to the dietary guidelines, intake of fruit and vegetables, dietary fibres, fish should be increased to receive positive score (adequate components) whereas intakes of the remaining five components (moderation components: red meat, SFA, sodium, SSB, and added sugar) should be decreased to receive positive score. Specific cut-off values for the components fruits and vegetables, fish, red meat, dietary fibre and SSB were derived from*

		the FBDG. For the nutrients, cut-off values were derived from the Nordic Nutrition Recommendations. The eight component scores were summed up to a total score ranging from zero to 80 points.<br><br>*Methods: The following variables, retrieved from interviews in GW12 and GW30, were included as covariates: maternal age (continuous and groups:≤25y, 26-30y, 31-35y, ≥36y), maternal pre-pregnancy BMI (underweight BMI < 18.5 kg/m2, normal weight BMI: 18.5-24.9 kg/m^2, overweight BMI: 25-29.9 kg/m^2, obese BMI ≥ 30 kg/m2), parity (0, 1, 2+ children), physical activity during pregnancy (low: < 4h/w, high: ≥ 4h/w), alcohol use during pregnancy (yes/no), smoking during pregnancy (never, occasionally, daily), lactation (≤ 1 month (m), 2-6m, ≥ 7m), parental educational level (high: more than 4 years of post-secondary education corresponding to a Master's or PhD degree; medium: corresponding to 3-4 years of post-secondary education (corresponding to Bachelor's degree); skilled worker: corresponding to nine years of basic school plus five years of vocational training; and finally unskilled worker).*
Data sources/ measurement	8*	For each variable of interest, give sources of data and details of methods of assessment (measurement). Describe comparability of assessment methods if there is more than one group<br>*Methods: The maternal FFQ completed during pregnancy was based on a questionnaire from the Danish Cancer Registry. The FFQ addressed ~360 food items and supplement use during the previous four weeks. Answer options ranged from 'never' to '8 or more times per week'. The FFQ was previously validated against 7-day food diaries and urine nitrogen excretion (n = 88). The FFQ at age 14 years was developed based on the validated FFQ used in the Growing Up Today Study and was completed by the offspring. The offspring FFQ addressed ~158 food items as well as questions about physical activity and supplement use during the previous year. Answer options ranged from 'did not drink/consume the last year' to '4 times or more per day'. For all food items in the maternal and in the offspring FFQ, standard portion sizes and in-house developed mixed dished recipes were applied. Frequency of intake was computed into grams per day using the nutrition software package FoodCalc v.3 combined with the Danish food table.*
Bias	9	Describe any efforts to address potential sources of bias<br>*Statistical analyses:*<br>*To adjust for potential confounding two models were applied: in **model A**) RR was adjusted for maternal age, pre-pregnancy BMI, parity, education, lactation, physical activity level, alcohol use, and smoking during pregnancy. In **model B**, additional adjustment was made for offspring sex and total energy intake of offspring (quartiles) to account for any potential correlation between offspring HEI score and energy intake.*<br>Page 13, statistical analyses:<br>*To evaluate potential selection bias due to cohort attrition, we compared maternal characteristics of the offspring who completed the FFQ at age 14 (n = 19,582) with those invited who did not complete the FFQ at 14 years of age (n = 29,433).*

		Statistical analyse:<br>*To take diseases potentially affecting eating behavior among women e.g. anorexia and inflammatory bowel disease which would result in very low energy intakes and extremely low BMI into consideration, we did several sensitivity analyses applied in Model B; a) excluding women with missing or very low BMI (≤18.5 kg/m^2, n = 517), b) excluding women with low energy intakes (<6.0 MJ/d, n = 1,723), c) excluding vegan/vegetarian mothers (n = 249). The HEI score as continuous variable was applied in a sensitivity analyses in the fully adjusted model B.*<br><br>*In a sub-sample of 595 mother-offspring pairs, mother's diet was assessed concurrently with offspring diet 14 years after pregnancy. In this sample we examined triangular associations between mother's HEI at pregnancy and mother-offspring HEI at 14 year follow-up. This was done by adjusting the main analysis (model B) for mother's HEI score assessed 14 years after pregnancy (S5 Text).*
Study size	10	Explain how the study size was arrived at<br>*Page 7, methods:*<br><br>*For the present study, offspring with dietary data collected from 2012 until fall 2015 (n ~ 24,000) and corresponding mothers (n = 68,240) whose maternal pregnancy dietary intake was available were included and matched into 21,082 mother-offspring pairs (Fig. 1). No exclusion criteria were set in terms of health status. However, we excluded implausible total energy intakes (< 2,500 kJ/day and > 25,000 kJ/day) (n = 351), multiple pregnancies and births (n = 1,144), and age ≥15 years (n =5) which resulted in a total of 19,582 mother-offspring pairs.*
Quantitative variables	11	Explain how quantitative variables were handled in the analyses. If applicable, describe which groupings were chosen and why<br>*Statistical analyses: In primary analyses we divided mother and offspring HEI score into quartiles*<br>*Statistical analyses: Covariates. maternal age (continuous and groups:≤25y, 26-30y, 31-35y, ≥36y), maternal pre-pregnancy BMI (underweight BMI < 18.5 kg/m2, normal weight BMI: 18.5-24.9 kg/m2, overweight BMI: 25-29.9 kg/m2, obese BMI ≥ 30 kg/m2), parity (0, 1, 2+ children), physical activity during pregnancy (low: < 4h/w, high: ≥ 4h/w), alcohol use during pregnancy (yes/no), smoking during pregnancy (never, occasionally, daily), lactation (≤ 1 month (m), 2-6m, ≥ 7m), parental educational level (high: more than 4 years of post-secondary education corresponding to a Master's or PhD degree; medium: corresponding to 3-4 years of post-secondary education (corresponding to Bachelor's degree); skilled worker: corresponding to nine years of basic school plus five years of vocational training; and finally unskilled worker).*
Statistical methods	12	(*a*) Describe all statistical methods, including those used to control for confounding<br>*Statistical analyses: We used a log-linear binomial model to estimate risk ratio (RR) and 95% confidence intervals (CI) for the offspring in the highest quartile of HEI with respect to maternal diet quality as*

		assessed by HEI. Analyses were performed using PROC GENMOD in SAS (SAS 9.4, Cary, NC). Because having a high HEI indicates having a more optimal diet according to dietary guidelines, we denoted relative chance as risk ratio. When testing for linear trend, the quartile variable was coded with the median HEI value in each quartile and entered as a continuous term to calculate p for trend.
		(*b*) Describe any methods used to examine subgroups and interactions<br>*Statistical analyses:*<br>*To examine potential sex differences in offspring we tested for interaction followed by stratified analyses.*<br><br>*To evaluate potential selection bias due to cohort attrition, we compared maternal characteristics of the offspring who completed the FFQ at age 14 (n = 19,582) with those invited who did not complete the FFQ at 14 years of age (n = 29,433).*<br>*Statistical analyse:*<br>*To take diseases potentially affecting eating behavior among women e.g. anorexia and inflammatory bowel disease which would result in very low energy intakes and extremely low BMI into consideration, we did several sensitivity analyses applied in Model B; a) excluding women with missing or very low BMI (≤18.5 kg/m^2, n = 517), b) excluding women with low energy intakes (<6.0 MJ/d, n = 1,723), c) excluding vegan/vegetarian mothers (n = 249). The HEI score as continuous variable was applied in a sensitivity analyses in the fully adjusted model B.*<br><br>*In a sub-sample of 595 mother-offspring pairs, mother's diet was assessed concurrently with offspring diet 14 years after pregnancy. In this sample we examined triangular associations between mother's HEI at pregnancy and mother-offspring HEI at 14 year follow-up. This was done by adjusting the main analysis (model B) for mother's HEI score assessed 14 years after pregnancy (S5 Text).*
		(*c*) Explain how missing data were addressed<br>*Statistical analyses:*<br><br>*Missing values for covariates ranged from 0.02% (maternal age groups) to 4.7% (pre-pregnancy BMI). Due to relatively low percentage of missing values (<5%), missing values for maternal age were imputed using the median values; while smoking, physical activity and parity were imputed using mode. For pre-pregnancy BMI, which had the highest frequency of missing, missing values were assigned to a separate missing category.*
		(*d*) If applicable, explain how loss to follow-up was addressed. *N.a.*
		(*e*) Describe any sensitivity analyses *Please see #9 and #12b*
**Results**		
Participants	13*	(a) Report numbers of individuals at each stage of study—eg numbers potentially eligible, examined for eligibility, confirmed eligible, included in the study, completing follow-up, and analysed<br>*Page 7, methods:*<br><br>*For the present study, offspring with dietary data collected from 2012 until fall 2015 (n ~ 24,000) and corresponding mothers (n = 68,240)*

		whose maternal pregnancy dietary intake was available were included and matched into 21,082 mother-offspring pairs (Fig. 1). No exclusion criteria were set in terms of health status. However, we excluded implausible total energy intakes (< 2,500 kJ/day and > 25,000 kJ/day) (n = 351), multiple pregnancies and births (n = 1,144), and age ≥15 years (n =5) which resulted in a total of 19,582 mother-offspring pairs.
		(b) Give reasons for non-participation at each stage N.A.
		(c) Consider use of a flow diagram Added as Fig.1.
Descriptive data	14*	(a) Give characteristics of study participants (eg demographic, clinical, social) and information on exposures and potential confounders<br>*Results, Study participants:*<br>*The mean age (±standard deviation [SD]) among mothers was 30.7 (±4.1) years, the majority of mothers were classified as normal-weight (67 %) and did not smoke during pregnancy (79 %). When dividing the population according to maternal HEI score during pregnancy, there was a significant difference across quartiles (Q) of the HEI score for all maternal characteristics. Most of the offspring were classified as normal-weight (76 %). The prevalence of offspring overweight was slightly higher in the lowest two quartiles of maternal HEI score*
		(b) Indicate number of participants with missing data for each variable of interest<br>*Table 2*
		(c) Summarise follow-up time (eg, average and total amount) *N.A*
Outcome data	15*	Report numbers of outcome events or summary measures over time *N.A.*
Main results	16	*(a) Give unadjusted estimates and, if applicable, confounder-adjusted estimates and their precision (e.g. 95% confidence interval). Make clear which confounders were adjusted for and why they were included*<br>*Please see #9for confounders*<br>*Results: The RR of the offspring with a high HEI score was 2-fold increased when comparing the highest to the lowest maternal quartile (RR: 2.1, 95%CI: 1.9, 2.3). After adjustment for maternal characteristics (model A) and additionally offspring sex and total energy intake (model B) the RR estimates were only slightly attenuated and remained significant (RR: 1.9, 95%CI: 1.8, 2.1).*<br>*Reason included for confounders in the Method section: Covariates: Mother's age, educational level, BMI, and lactation are known predictors of offspring dietary habits. Parity, physical activity, alcohol use, and smoking were included in order to adjust for potential confounding due to social and behavioral factors related to offspring lifestyle.*
		(*b*) Report category boundaries when continuous variables were categorized
		(*c*) If relevant, consider translating estimates of relative risk into absolute risk for a meaningful time period N.A.
Other analyses	17	Report other analyses done—eg analyses of subgroups and interactions, and sensitivity analyses<br>*Results:*<br>*When we compared maternal characteristics for those offspring who completed the FFQ14 (n = 19,582) with those invited who did not complete the FFQ14 (n = 29,433) we found that those who completed the FFQ14 had significantly older mothers (mean difference [95% CI]:*

0.59 years [0.51, 0.67] p < 0.001), mothers had lower pre-pregnancy BMI (mean difference [95% CI]: -0.49 kg/m^2 [-0.57, 0.41], p < 0.001), and a higher proportion had a higher educational level (25 % vs 20 %, p < 0.001). We observed no significant difference for level of parity (49 % vs 48 % had no prior children, p = 0.20). The proportion of girls was significantly different between adolescents who did or did not complete the FFQ14, respectively (52 % vs 46 %, p < 0.001) (S2 Table).

*Results: As expected maternal and offspring intake of fruit and vegetables, dietary fibre, and fish increased with increasing HEI scores, while the opposite was observed for red meat (mothers only), SFA, SSB and added sugars (Table 3). However, no trend was observed for sodium and total energy intake across the HEI quartiles. For sodium and energy intake we additionally observed essentially no correlation between offspring intake and offspring HEI score (r = -0.03 and 0.06, respectively) (**Table 4**). The maternal HEI score was correlated positively with offspring HEI score (Pearson r = 0.22, p < 0.001).*

Results: In table 5, results were presented based on categorizing offspring and maternal HEI into quartiles. When examining the association between maternal offspring HEI on a continuous scale per 10-unit increase of maternal HEI score during pregnancy was associated with 2.3 (95%CI: 2.1, 2.4) unit increase in offspring HEI score 14 years later. The maternal HEI score explained around 14% of the variance in the offspring HEI score.

Results: Despite almost 2-fold increase in RR for the offspring being in the highest HEI quartile if the mother was in the highest quartile during pregnancy, the variation in offspring HEI within each maternal quartile was substantial and overlapping with adjacent quartiles (S4 Fig.). We observed no change in direction or magnitude in the sex stratified, fully adjusted analyses (Model B) for boys (RR: 1.9, 95%CI: 1.7, 2.3) or girls (RR: 1.9, 95%CI: 1.8, 2.2), respectively (S5A Table).
*In secondary analyses, we examined further potential modification by maternal characteristics in stratified analyses by maternal pre-pregnancy BMI (**Table 6**), parental education and smoking during pregnancy (S5B Table). Risk ratio estimates were comparable to and in the same direction as those from the non-stratified analyses (as presented in Table 5). Furthermore, to evaluate if one HEI component was responsible for the association between maternal offspring diet, we added one component of maternal HEI at a time to the model B (S6 Table). The results were within the same direction as the results presented in Table 5 and no specific component appeared to be responsible for the relationship between maternal and offspring HEI scores).*
Page 24, results: Adjustment for maternal HEI score assessed at 14 years after pregnancy had limited impact on the association between maternal diet quality in pregnancy and offspring diet quality at 14 years. The RR of 1.9 (95%CI: 1.8, 2.1) was a very similar estimate as obtained for the full sample without adjustment in Table 5.

**Discussion**		
Key results	18	Summarise key results with reference to study objectives

		Discussion: In this study of ~20,000 mother-child pairs we found that offspring's diet quality was positively associated to its mother's antenatal diet quality, despite the fact that the two dietary assessments were undertaken 15 years apart.
Limitations	19	Discuss limitations of the study, taking into account sources of potential bias or imprecision. Discuss both direction and magnitude of any potential bias<br>*Discussion*: *A number of limitations within the study are acknowledged. The FFQ method has some limitations as it relies on the participant's ability to recall and report dietary intake, and calculation relies on the content of the applied food table. If memory-bias affected accuracy of assessed dietary intake equally among participants, level of diet quality might have been skewed in either direction compared with true intake, which would not affect ranking across groups. A registry based study conducted at the time found no indication of attrition bias with respect to maternal smoking and adverse pregnancy outcomes such as preterm delivery and low birth weight (<2500g) [36] . However, as with all longitudinal studies with long term follow-up there is substantial attrition. In our 20 year follow-up, proximately 50% of those invited agreed to participate which is not unexpected among young adults.*<br>*We did not have the possibility to adjust for the potential confounding of e.g. unhealthy habits such as smoking and socio-economic status of the mother, which could have changed systematically after pregnancy. Confounding by later environmental exposures could occur if these were somehow related to changes in dietary habits. However, we would still think it is more likely that socio-economic and healthy lifestyle habits would remain fairly constant for most subjects. In any case, the positive association we observe is more in the direction that one would expect rather than the opposite.*
Interpretation	20	Give a cautious overall interpretation of results considering objectives, limitations, multiplicity of analyses, results from similar studies, and other relevant evidence<br>*Discussion: Irrespective of which mechanisms may be underlying the observed associations between characteristics of the mother's diet in pregnancy and the offspring's diet many years later, our findings have relevant implications for studies focusing on environmental factors operating during the years of development on aetiologies of adult diseases. Our results emphasise the need for taking offspring diet quality into account when studying the association between dietary factors in pregnancy and offspring adult health, and – vice versa – the need for taking dietary factors in pregnancy into account when studying associations between dietary exposures in adolescent years and adult health.*
Generalisability	21	Discuss the generalisability (external validity) of the study results<br>*Discussion: The analysis on participants vs. non-participants in the FFQ14 follow-up study indicated some level of attrition bias towards healthier participants and higher socio-economic status. The implication by those with poorer diet quality might be underrepresented in our study population, but at the same time it is not clear how such bias could lead to spurious correlation between maternal offspring dietary habits. We did not have the possibility to adjust for the potential confounding of e.g. unhealthy habits such as smoking and socio-*

		economic status of the mother, which could have changed systematically after pregnancy. Confounding by later environmental exposures could occur if these were somehow related to changes in dietary habits. However, we would still think it is more likely that socio-economic and healthy lifestyle habits would remain fairly constant for most subjects. In any case, the positive association we observe is more in the direction that one would expect rather than the opposite.
**Other information**		
Funding	22	Give the source of funding and the role of the funders for the present study and, if applicable, for the original study on which the present article is based<br>*This work was supported by grants from The Danish Research Council now: Independent Research Fund Denmark (www.dff.dk) [09-067124 (Centre for Fetal Programming)] and [09-075611]; and by Nordea-fonden (www.nordeafonde.dk) [02-2013-2014] to SFO. The funders had no role in study design, data collection and analysis, decision to publish, or preparation of the manuscript.*

*Give information separately for exposed and unexposed groups.

Note: An Explanation and Elaboration article discusses each checklist item and gives methodological background and published examples of transparent reporting. The STROBE checklist is best used in conjunction with this article (freely available on the Web sites of PLoS Medicine at http://www.plosmedicine.org/, Annals of Internal Medicine at http://www.annals.org/, and Epidemiology at http://www.epidem.com/). Information on the STROBE Initiative is available at http://www.strobe-statement.org.
