# Supplementary material for: Mother’s dietary quality during pregnancy and offspring’s dietary quality in adolescence: Follow-up from a national birth cohort study of 19,582 mother–offspring pairs
Source: PLoS Med. 2019 Sep 12;16(9):e1002911. doi: 10.1371/journal.pmed.1002911 (PMC6742222; doi:10.1371/journal.pmed.1002911)
Supplement: S1 Table — FFQ, food frequency questionnaire. (PDF) [file pmed.1002911.s002.pdf]

Article title: Mother's dietary quality during pregnancy and offspring's dietary quality in adolescence: follow-up from a nationwide birth cohort study of 19,582 mother-offspring pairs

Author names: Anne Ahrendt Bjerregaard, Thorhallur Ingi Halldorsson, Inge Tetens, Sjurdur Frodi Olsen

Affiliation and e-mail address of corresponding author

Center for Fetal Programming, Department of Epidemiology Research, Statens Serum Institut, Copenhagen, Denmark, anne@ssi.dk

S1 Table. Analyses of attrition

A Comparison of maternal characteristics for those offspring who completed the FFQ14 (*n* = 19,582) and maternal characteristics for those invited who did not complete the FFQ14 (*n* = 29,433).

	**Main study population**	**Non-participants**	
	***n* = 19,582**	***n* = 29,433**	***p****
**Maternal characteristics**	Mean (±SD) or %		
Age groups (years)	30.7 (4.1)	30.1 (4.4)	<**0.001**
Pre-pregnancy BMI [1]	23.3 (3.9)	24.4 (4.4)	<**0.001**
Smoking during pregnancy			<**0.001**
No	79	70	
Yes Occasional	20	18	
Missing	1	3	
Parity			0.20
No prior child	49	48	
Prior children	51	52	
Parental education			<**0.001**
High	25	20	
Medium	32	27	
Skilled workers	25	29	
Unskilled/students/ unemployed	18	24	
Proportion of offspring girls	53	46	**<0.001**

*One-way ANOVA for continuous and chi-squared test for categorical variables, respectively.

B Relative risk estimates from three sensitivity analyses excluding women with characteristic that may affect eating behaviour; a) low maternal BMI excluded (*n* = 17,859), b) maternal low energy intake excluded (*n* = 19,065), and c) maternal vegan/vegetarian excluded (*n* = 19,333)

	**Population a**	**Population b**	**Population c**	
	BMI > 18.5 kg/m^2	Maternal energy intake >6.0 MJ/d	No vegan/vegetarian mothers	
	***n* = 17,859**	***n* = 19,065**	***n* = 19,333**	***p****
Maternal HEI quartiles		RR model B[a]		
Q1	1	1	1	
Q2	1.17 (1.29, 1.42)	1.18 (1.30, 1.42)	1.18 (1.29, 1.42)	<0.001
Q3	1.37 (1.51, 1.65)	1.40 (1.53, 1.67)	1.41 (1.54, 1.69)	<0.001
Q4	1.76 (1.92, 2.10)	1.81 (1.97, 2.15)	1.81 (1.97, 2.15)	<0.001

[a]Adjusted for maternal age, pre-pregnancy BMI, parity, education, physical activity, smoking and alcohol intake during pregnancy, lactation, offspring energy intake and sex. * Statistically significance in all three populations
