# Supplementary material for: Mother’s dietary quality during pregnancy and offspring’s dietary quality in adolescence: Follow-up from a national birth cohort study of 19,582 mother–offspring pairs
Source: PLoS Med. 2019 Sep 12;16(9):e1002911. doi: 10.1371/journal.pmed.1002911 (PMC6742222; doi:10.1371/journal.pmed.1002911)
Supplement: S2 Table — (PDF) [file pmed.1002911.s003.pdf]

Article title: Mother's dietary quality during pregnancy and offspring's dietary quality in adolescence: follow-up from a nationwide birth cohort study of 19,582 mother-offspring pairs

Author names: Anne Ahrendt Bjerregaard, Thorhallur Ingi Halldorsson, Inge Tetens, Sjurdur Frodi Olsen

Affiliation and e-mail address of corresponding author

Center for Fetal Programming, Department of Epidemiology Research, Statens Serum Institut, Copenhagen, Denmark, anne@ssi.dk

S2 Table. Stratified analyses

A Maternal prenatal dietary habits as predictor of dietary habits offspring **BOYS** (*n* = 9,289) and **GIRLS** (*n* = 10,293).

BOYS				
Maternal HEI quartiles	Cases[1]/N	RR	RR model A[2]	RR model B[3]
Q1	452/2,904	1	1	1
Q2	572/2,505	1.33 (1.15, 1.54)	1.29 (1.12, 1.50)	1.29 (1.10, 1.51)
Q3	664/2,209	1.64 (1.43, 1.89)	1.57 (1.36, 1.80)	1.54 (1.32, 1.80)
Q4	584/1,671	2.20 (1.93, 2.51)	2.01 (1.76, 2.31)	1.98 (1.70, 2.30)
p for trend		<0.001	<0.001	<0.001
GRILS				
Maternal HEI quartiles				
Q1	301/1,958	1	1	1
Q2	471/2,281	1.31 (1.19, 1.44)	1.28 (1.16, 1.41)	1.29 (1.15, 1.44)
Q3	671/2,715	1.56 (1.42, 1.71)	1.49 (1.35, 1.63)	1.50 (1.35, 1.68)
Q4	1142/3,239	2.09 (1.92, 2.28)	1.95 (1.78, 2.13)	1.95 (1.76, 2.17)
p for trend		<0.001	<0.001	<0.001

[1] Cases=the number of offspring in Q4 according to ranking of the mother
[2]Adjusted for maternal age, pre-pregnancy BMI, parity, education, physical activity, smoking and alcohol intake during pregnancy, lactation
[3]Additionally adjusted for offspring energy intake and BMI
HEI, healthy eating index; Q, quartile

B Strata analyses of maternal prenatal dietary habits as predictor of 14y offspring dietary habits (*n* = 19,582)

Maternal HEI quartiles	Cases[1]/N	RR	RR model A[2]	RR model B[3]
Parental education				
High				
Q1	199/979	1	1	1
Q2	313/1,155	1.33 (1.12, 1.58)	1.32 (1.12, 1.57)	1.36 (1.15, 1.60)
Q3	404/1,329	1.59 (1.36, 1.87)	1.56 (1.33, 1.83)	1.54 (1.32, 1.80)
Q4	564/1,419	2.04 (1.75, 2.37)	1.96 (1.68, 2.28)	1.95 (1.67, 2.26)
p for trend		<0.001	<0.001	<0.001
Medium				
Q1	284/1,627	1	1	1
Q2	384/1,796	1.28 (1.12, 1.47)	1.26 (1.10, 1.45)	1.28 (1.12, 1.46)
Q3	514/1,809	1.58 (1.39, 1.80)	1.54 (1.35, 1.75)	1.54 (1.35, 1.75)
Q4	679/1,856	2.09 (1.85, 2.36)	1.98 (1.75, 2.24)	1.96 (1.74, 2.21)
p for trend		<0.001	<0.0001	<0.0001
Low				
Q1	169/1,482	1	1	1
Q2	204/1,217	1.29 (1.09, 1.53)	1.27 (1.07, 1.51)	1.26 (1.07, 1.49)
Q3	239/1,123	1.56 (1.32, 1.84)	1.53 (1.29, 1.80)	1.49 (1.26, 1.75)
Q4	282/986	2.21 (1.89, 2.58)	2.10 (1.79, 2.47)	2.04 (1.75, 2.39)
p for trend		<0.001	<0.001	<0.001
Smoking during pregnancy				
No				
Q1	617/3,720	1	1	1
Q2	867/3,877	1.31 (1.19, 1.44)	1.27 (1.16, 1.40)	1.28 (1.17, 1.40)
Q3	1088/3,910	1.58 (1.44, 1.73)	1.52 (1.38, 1.66)	1.50 (1.38, 1.64)
Q4	1458/3,918	2.19 (2.10, 2.38)	2.02 (1.85, 2.20)	1.99 (1.83, 2.16)
p for trend		<0.001	<0.001	<0.001
Yes				
Q1	136/1,142	1	1	1
Q2	176/1,009	1.33 (1.13, 1.57)	1.28 (1.09, 1.51)	1.33 (1.13, 1.56)
Q3	247/1,014	1.61 (1.37, 1.89)	1.51 (1.29, 1.78)	1.53 (1.30, 1.79)
Q4	268/992	1.92 (1.64, 2.24)	1.78 (1.51, 2.08)	1.77 (1.51, 2.07)
p for trend		<0.001	<0.001	<.0.001

[1] Cases=the number of offspring in Q4 according to ranking of the mother [2]Adjusted for maternal age, pre-pregnancy BMI, parity, education, physical activity, smoking and alcohol intake during pregnancy, lactation. [3]Additionally adjusted for offspring energy intake and sex. HEI, healthy eating index; Q, quartile
