# Supplementary material for: Mother’s dietary quality during pregnancy and offspring’s dietary quality in adolescence: Follow-up from a national birth cohort study of 19,582 mother–offspring pairs
Source: PLoS Med. 2019 Sep 12;16(9):e1002911. doi: 10.1371/journal.pmed.1002911 (PMC6742222; doi:10.1371/journal.pmed.1002911)
Supplement: S3 Table — HEI, Healthy Eating Index. (PDF) [file pmed.1002911.s004.pdf]

Article title: **M**other’s dietary quality during pregnancy and offspring’s dietary quality in adolescence: follow-up from a nationwide birth cohort study of 19,582 mother-offspring pairs

Author names: Anne Ahrendt Bjerregaard, Thorhallur Ingi Halldorsson, Inge Tetens, Sjurdur Frodi Olsen

Affiliation and e-mail address of corresponding author

Center for Fetal Programming, Department of Epidemiology Research, Statens Serum Institut, Copenhagen, Denmark, anne@ssi.dk

S3 Table. Adjustment for the individual HEI components’

The table includes results on the association between maternal prenatal dietary habits as predictor of 14y offspring dietary habits additionally adjusted for individual healthy eating index components.

Maternal prenatal dietary habits as predictor of dietary habits offspring adjusted for individual HEI components (*n* = 19,582).

Maternal HEI quartiles	RR model B, Table 5[a]	Relative Risk							
		Fruit & vegetables[b]	Dietary fibres[b]	Fish[b]	Red Meat[b]	Saturated fatty acids[b]	Sodium[b]	SSB[b]	Added sugar[b]
Q1	1	1	1	1	1	1	1	1	1
Q2	1.29 (1.18, 1.42)	1.14 (1.25,1.37)	1.17 (1.29, 1.41)	1.15 (1.27, 1.39)	1.16 (1.27, 1.40)	1.17 (1.28, 1.40)	1.18 (1.29, 1.41)	1.18 (1.29, 1.41)	1.18 (1.29, 1.42)
Q3	1.53 (1.40, 1.67)	1.31 (1.44, 1.54)	1.39 (1.52, 1.66)	1.35 (1.48, 1.63)	1.36 (1.49, 1.63)	1.36 (1.49, 1.63)	1.39 (1.52, 1.67)	1.39 (1.52, 1.67)	1.40 (1.53, 1.68)
Q4	1.97 (1.81, 2.15)	1.66 (1.83, 2.00)	1.80 (1.96, 2.15)	1.73 (1.90, 2.09)	1.72 (1.89, 2.08)	1.73 (1.89, 2.06)	1.81 (1.98, 2.15)	1.81 (1.97, 2.16)	1.80 (1.98, 2.18)

[a] RR model B, Table 5 is adjusted for maternal age, pre-pregnancy BMI, parity, education, physical activity, smoking and alcohol intake during pregnancy, lactation, offspring energy intake, and gender

[b] RR model B, Table 5 additionally adjusted for this HEI component

HEI, healthy eating index; Q, quartile; RR, relative risk; SSB, sugar sweetened beverages
