# Supplementary material for: Mother’s dietary quality during pregnancy and offspring’s dietary quality in adolescence: Follow-up from a national birth cohort study of 19,582 mother–offspring pairs
Source: PLoS Med. 2019 Sep 12;16(9):e1002911. doi: 10.1371/journal.pmed.1002911 (PMC6742222; doi:10.1371/journal.pmed.1002911)

Article title: Mother's dietary quality during pregnancy and offspring's dietary quality in adolescence: follow-up from a nationwide birth cohort study of 19,582 mother-offspring pairs

Author names: Anne Ahrendt Bjerregaard, Thorhallur Ingi Halldorsson, Inge Tetens, Sjurdur Frodi Olsen

Affiliation and e-mail address of corresponding author

Center for Fetal Programming, Department of Epidemiology Research, Statens Serum Institut, Copenhagen, Denmark, e-mail: anne@ssi.dk

S1 Figure.

Offspring median HEI score (25th – 75th percentile) as a function of maternal HEI score in quartiles. The HEI score could range from zero to 80 points.

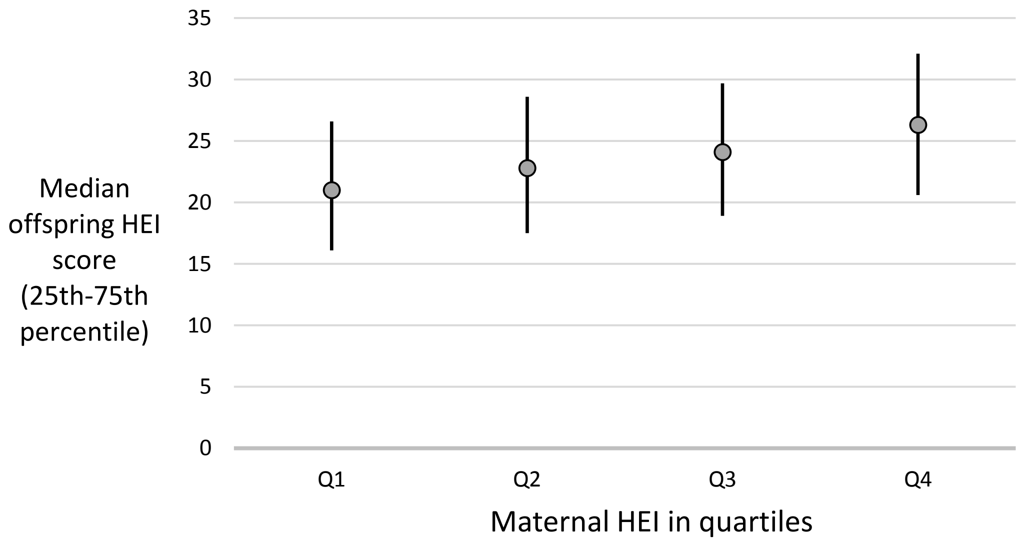

Supplement: S1 Fig — HEI, Healthy Eating Index. (PDF) [file pmed.1002911.s005.pdf]
