# Supplementary material for: Mother’s dietary quality during pregnancy and offspring’s dietary quality in adolescence: Follow-up from a national birth cohort study of 19,582 mother–offspring pairs
Source: PLoS Med. 2019 Sep 12;16(9):e1002911. doi: 10.1371/journal.pmed.1002911 (PMC6742222; doi:10.1371/journal.pmed.1002911)
Supplement: S1 Appendix — HEI, Healthy Eating Index. (PDF) [file pmed.1002911.s006.pdf]

Article title: Mother's dietary quality during pregnancy and offspring's dietary quality in adolescence: follow-up from a national birth cohort study of 19,582 mother-offspring pairs

Author names: Anne Ahrendt Bjerregaard, Thorhallur Ingi Halldorsson, Inge Tetens, Sjurdur Frodi Olsen

Affiliation and e-mail address of corresponding author
Center for Fetal Programming, Department of Epidemiology Research, Statens Serum Institut, Copenhagen, Denmark, anne@ssi.dk

S1 Appendix. Sub-sample analyses

Sub-sample analyses includes description of and results from triangular association analyses between mother─offspring HEI at pregnancy and at 14 years follow-up

A Text Background, methods, and results of the triangular association analyses

With increased emphasis on obtaining more detailed information on nutritional exposure in cohort studies, we set out to examine whether asking one or both parents to fill out an additional FFQs would affect the response rate of the offspring at a 14 year follow-up within the Danish National Birth Cohort (DNBC). With the design of a randomized controlled trial (RCT) and an online questionnaire, it was possible to determine the effect from parental participation on adolescent response rate in a highly controlled setting. The RCT was initiated within the DNBC 14 year follow-up from January to May 2013. Singleton offspring (n = 5,829) who had not taken part in the 14-year-old follow-up at the time of initiating the RCT, were identified as eligible as they were living with both parents, or living with mother and father was accessible at another address. The adolescents were randomised into three 'invitation'-groups (n=1,942 in each group):

A) Adolescents only,

B) Adolescent plus mother,

C) Adolescent plus mother and father.

The invitations were send by email when possible otherwise, the invitations were send by postal letter. Up to three reminders were administered to increase response rate.

We used the sub-set of DNBC participants from 'invitation'-group B to examine the triangular dietary relationship, in terms of healthy eating index (HEI), between (1) mother's diet during pregnancy, (2)

her own diet at the 14 year follow-up and (3) the relationship between these two time points with offspring own diet at 14 years. The overall aim was to explore if our estimates presented in Table 5 in the main manuscript, would be affected when taking into consideration mother own diet at the 14 year follow-up. After exclusion of implausible energy intakes (< 2,500 kJ/day and > 25,000 kJ/day) 595 mother-offspring pairs with a HEI score assessment 14 years after birth were eligible. These were compared with the mothers from the full study sample, who were not part of the sub-study (*n*=18,987)

Results

Comparison of maternal and offspring characteristics, respectively between the two study populations are presented in **Table 1 and 2**. Mothers in the sub-study population (*n* = 595) were more likely to have more children ($p < 0.001$) compared to the remaining mothers from the full study population (*n* = 18,987). No differences were observed for offspring characteristics. The mean HEI score (SD) in the sub-sample was 26 (8) points for mothers 14 years after pregnancy. This was significantly higher compared with the mothers mean HEI score (mothers: 23 (7) points) in the main study population ($p < 0.001$). No significant differences were observed in the mean HEI scores [SD] among offspring in the RCT and in the main study (23.5 [8.7] points vs. 24.2 [8.5] points).

We found that maternal prenatal and postnatal HEI score was significantly correlated with each other and with offspring HEI score (**Figure 1**). The risk ratio (RR) estimates were in the same direction as in the full population analyses. Further adjustment for maternal postnatal HEI score at the 14 year follow-up resulted in a reduction in RR from 2.54 to 1.84 (**Table 3**).

B Table Maternal characteristics

	Main study population	Sub-study population	
	***n* = 18,987**	***n* = 595**	***p****
**Maternal characteristics**	Mean (SD) or %		
Age groups (years)	30.7 (4.1)	31.0 (3.9)	0.08
≤25	12	10	0.13
26-30	43	40	
31-35	34	39	
≥36	11	11	
Pre-pregnancy BMI [1]	23.3 (3.9)	23.0 (3.9)	0.11
Under weight	4	5	0.23
Normal weight	67	70	
Overweight and obese	24	22	
Missing	5	3	
Physical activity during pregnancy			0.09
No (<1h/week)	63	60	
Yes 1h or more/week	37	40	
Smoking during pregnancy			**0.046**
No	79	82	
Yes Occasional	20	17	
Missing	1	1	
Lactation			0.23
≤1m	31	29	
2-6m	20	18	
≥7m	49	53	
Parity			**<0.001**
No prior child	47	37	
Prior children	51	64	
Parental education			0.91
High	25	24	
Medium	36	37	
Skilled workers	25	24	
Unskilled/students/ unemployed	14	15	

*One-way ANOVA for continuous and chi-squared test for categorical variables, respectively.
[1]BMI body mass index Underweight BMI < 18.5 kg/m^2, normal weight BMI 18.5-24.9 kg/m^2, Over weight BMI 25-29.9 kg/m^2, obese BMI ≥ 30 kg/m^2

C Table Offspring characteristics

	Main population n=18,987	Sub-study population n=595	P*
**Offspring characteristics**	Mean (SD) or %		
Girls (%)	52.6	52.3	0.88
BMI [2]	19.2 (2.8)	19.2 (2.5)	0.87
Under weight	15	16	
Normal weight	76	75	
Overweight and obese	9	9	

BMI body mass index. *Anova for continuous and chi-squared for categorical variables.
[2]Child BMI: age and sex specific classified by Cole TJ, Bellizzi MC, Flegal KM, Dietz WH. Establishing a standard definition for child overweight and obesity worldwide: international survey. BMJ. 2000;320(7244):1240-3

D Figure Spearman correlation coefficients (*r*) between a) prenatal maternal and 14-year offspring HEI score ($r = 0.25$, $p < 0.001$); b) prenatal and postnatal maternal HEI score ($r = 0.37$, $p < 0.001$); and c) postnatal maternal and 14-year offspring HEI score ($r = 0.29$, $p < 0.001$). (*n*=595).

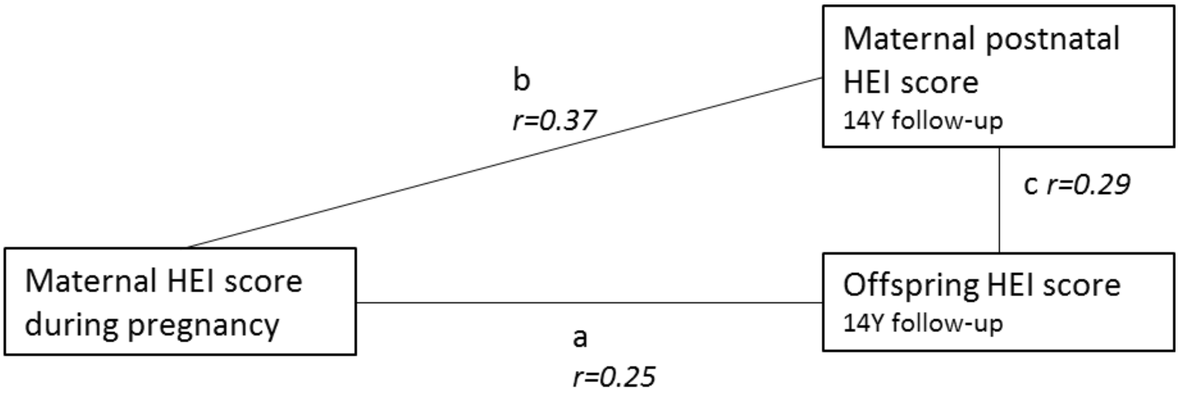

HEI: healthy eating index

E Table Association between maternal HEI during pregnancy and offspring HEI at 14Y before and after adjustment for mothers diet at 14 years (*n* = 595)

Maternal HEI quartiles	Cases[1]/N	RR (model B)[2]	RR (model C)[3]
Q1	23/146	1	1
Q2	29 /150	1.18 (0.68, 2.05)	1.06 (0.62, 1.84)
Q3	37 /153	1.58 (0.92, 2.69)	1.28 (0.74, 2.20)
Q4	60/147	2.54 (1.52, 4.24)	1.84 (1.08, 3.14)
p for trend		<0.001	<0.001

[1] Cases=the number of offspring in Q4 according to ranking of the mother

[2]Adjusted for maternal age, pre-pregnancy BMI, parity, education, physical activity, smoking and alcohol intake during pregnancy, lactation, offspring energy intake, offspring sex

[3]Additionally adjusted for maternal postnatal diet HEI score assessed concurrently with offspring 14y after birth

HEI, healthy eating index; Q, quartile
